# Dynamic Determinants of the Uncontrolled Manifold during Human Quiet Stance

**DOI:** 10.3389/fnhum.2016.00618

**Published:** 2016-12-06

**Authors:** Yasuyuki Suzuki, Hiroki Morimoto, Ken Kiyono, Pietro G. Morasso, Taishin Nomura

**Affiliations:** ^1^Department of Mechanical Science and Bioengineering, Graduate School of Engineering Science, Osaka UniversityOsaka, Japan; ^2^Robotics, Brain and Cognitive Sciences Department, Fondazione Istituto Italiano di TecnologiaGenoa, Italy

**Keywords:** posture control, uncontrolled manifold, intermittent control, postural sway, double inverted pendulum, postural stability

## Abstract

Human postural sway during stance arises from coordinated multi-joint movements. Thus, a sway trajectory represented by a time-varying postural vector in the multiple-joint-angle-space tends to be constrained to a low-dimensional subspace. It has been proposed that the subspace corresponds to a manifold defined by a kinematic constraint, such that the position of the center of mass (CoM) of the whole body is constant in time, referred to as the kinematic uncontrolled manifold (*kinematic-UCM*). A control strategy related to this hypothesis (*CoM-control-strategy*) claims that the central nervous system (CNS) aims to keep the posture close to the kinematic-UCM using a continuous feedback controller, leading to sway patterns that mostly occur within the kinematic-UCM, where no corrective control is exerted. An alternative strategy proposed by the authors (*intermittent control-strategy*) claims that the CNS stabilizes posture by intermittently suspending the active feedback controller, in such a way to allow the CNS to exploit a stable manifold of the saddle-type upright equilibrium in the state-space of the system, referred to as the *dynamic-UCM*, when the state point is on or near the manifold. Although the mathematical definitions of the kinematic- and dynamic-UCM are completely different, both UCMs play similar roles in the stabilization of multi-joint upright posture. The purpose of this study was to compare the dynamic performance of the two control strategies. In particular, we considered a double-inverted-pendulum-model of postural control, and analyzed the two UCMs defined above. We first showed that the geometric configurations of the two UCMs are almost identical. We then investigated whether the UCM-component of experimental sway could be considered as passive dynamics with no active control, and showed that such UCM-component mainly consists of high frequency oscillations above 1 Hz, corresponding to anti-phase coordination between the ankle and hip. We also showed that this result can be better characterized by an eigenfrequency associated with the dynamic-UCM. In summary, our analysis highlights the close relationship between the two control strategies, namely their ability to simultaneously establish small CoM variations and postural stability, but also make it clear that the intermittent control hypothesis better explains the spectral characteristics of sway.

## Introduction

Traditionally, the major origin of human postural sway during quiet stance has been considered to stem from rotational body motion around the ankle joints (Winter et al., 1998). However, accurate sway measurements in recent years have revealed that, along with ankle joint motion, several other joint movements (including the hip joints) are involved in postural sway (e.g., Aramaki et al., 2001; Creath et al., 2005; Hsu et al., 2007). It is now a common view that inter-joint coordination plays a substantial role in maintaining upright posture. In fact, the coordination of multiple joints during quiet stance can be considered as a natural extension of adaptive responses (e.g., ankle, hip, and mixed strategies) to external disturbances (Horak and Nashner, 1986). For example, Aramaki et al. (2001) measured ankle and hip joint motion during quiet stance and reported that ranges of angular rotation, velocity, and acceleration of the hip joint angle are comparable with, or even greater than, those of the ankle joint. Moreover, they revealed that angular acceleration of the ankle and hip joints is negatively correlated with each other at a specific ratio, suggesting that such specific coordination might reflect active control of the central nervous system (CNS) in minimizing the acceleration of the center of mass (CoM) position of the whole body. Sasagawa et al. (2009) simultaneously measured ankle and hip joint motion with the ground reaction force during quiet stance, and compared two different estimates of the CoM acceleration, one obtained as a linear combination of the ankle and hip joint accelerations and the other as the horizontal component of the ground reaction force divided by the body mass. Using this approach, they demonstrated that the two estimates are well matched, i.e., the latter estimate is characterized by the former with specific weight coefficients, implying indeed that hip joint motions make substantial contributions to neural control during quiet stance.

Because of the coordinated, multiple joint movements that occur during quiet stance, the posture of the multi-link body at every instance of time, represented by a vector in the multiple-joint angle space, is not distributed evenly in that space as it tends to be constrained to a specific low-dimensional space (Creath et al., 2005; Pinter et al., 2008). This fact indicates that the kinematic degrees of freedom of the human body during upright stance are redundant for achieving the goal of postural stability, and that control mechanisms that reduce the functional number of degrees of freedom must exist. Candidate mechanisms range from active neural feedback control including spinal reflexes and supra-spinal circuitry (e.g., see a recent review by Mori et al., 2016) to simple stiffness control, which is related to the passive characteristics of human biomechanics including mechanical impedance of muscles and joints, or a combination of the two. This study aimed to examine which mechanism was the most physiologically plausible.

Hsu et al. (2007) considered double- and six-link inverted pendulum models of upright posture during quiet stance (operating in the sagittal plane), and performed uncontrolled manifold (UCM) analysis (Scholz and Schöner, 1999) for the time-varying postural vector in the corresponding two- and six-dimensional joint angle spaces, respectively. Specifically, they defined UCMs (one- and five-dimensional hyperplanes in the two- and six-dimensional joint angle spaces, respectively) that satisfied the kinematic constraint of keeping the anterior-posterior position of the whole-body CoM fixed and constant in time, referred to here as *the kinematic-UCM*; subsequently, they analyzed experimental sway data assimilated to each of the models and decomposed the sway patterns into a component in the kinematic-UCM and a complemental component in the subspace orthogonal to the kinematic-UCM. Their analysis revealed that the postural vector mainly moves in the kinematic-UCM, relative to the orthocomplemental direction. Because joint motion in the kinematic-UCM does not cause changes in CoM position, this observation suggests that sway motion in the kinematic-UCM is generated when the CNS suspends interventional actions on postural control, whereas joint motions that cause changes in CoM position (i.e., sway motion in the orthocomplemental direction to the kinematic-UCM) are impeded by active control. Based on this interpretation, Hsu et al. claimed that the CNS stabilizes the position of the whole-body CoM using a neural feedback controller that forces the postural vector close to the kinematic-UCM, referred to here as *the CoM-control strategy or the CoM-control hypothesis*. In this case, the neural feedback controller is inactivated when the postural vector is located within the kinematic-UCM, whereby joint movement in the kinematic-UCM appears as the major component of postural sway.

Small variability in the whole-body CoM in the anterior-posterior direction implies that the two major body segments with large masses, namely the upper body (head-arms-trunk complex; HAT) and the lower extremities, tend to move in opposite directions (anti-phase coordination) rather than in the same direction (in-phase coordination). Indeed, quantitative analyses of motion-captured data for postural sway—assuming a double inverted pendulum model with HAT and lower extremity links connected by two joints (i.e., ankle and hip joints)—have shown that the two links exhibit in-phase coordination at low frequencies (below 1 Hz) and that anti-phase coordination occurs at high frequencies (between 1 and 5 Hz) (Creath et al., 2005; Zhang et al., 2007; Kato et al., 2014). Because anti-phase coordination contributes to a reduction of CoM-shift while in-phase coordination induces a CoM-shift, it is expected that the high- and low-frequency components of postural sway might correspond, respectively, to movements of the postural vector in the kinematic-UCM and those in the orthocomplemental subspace. In this study, we examined this expectation in a quantitative manner.

**Figure 1:**
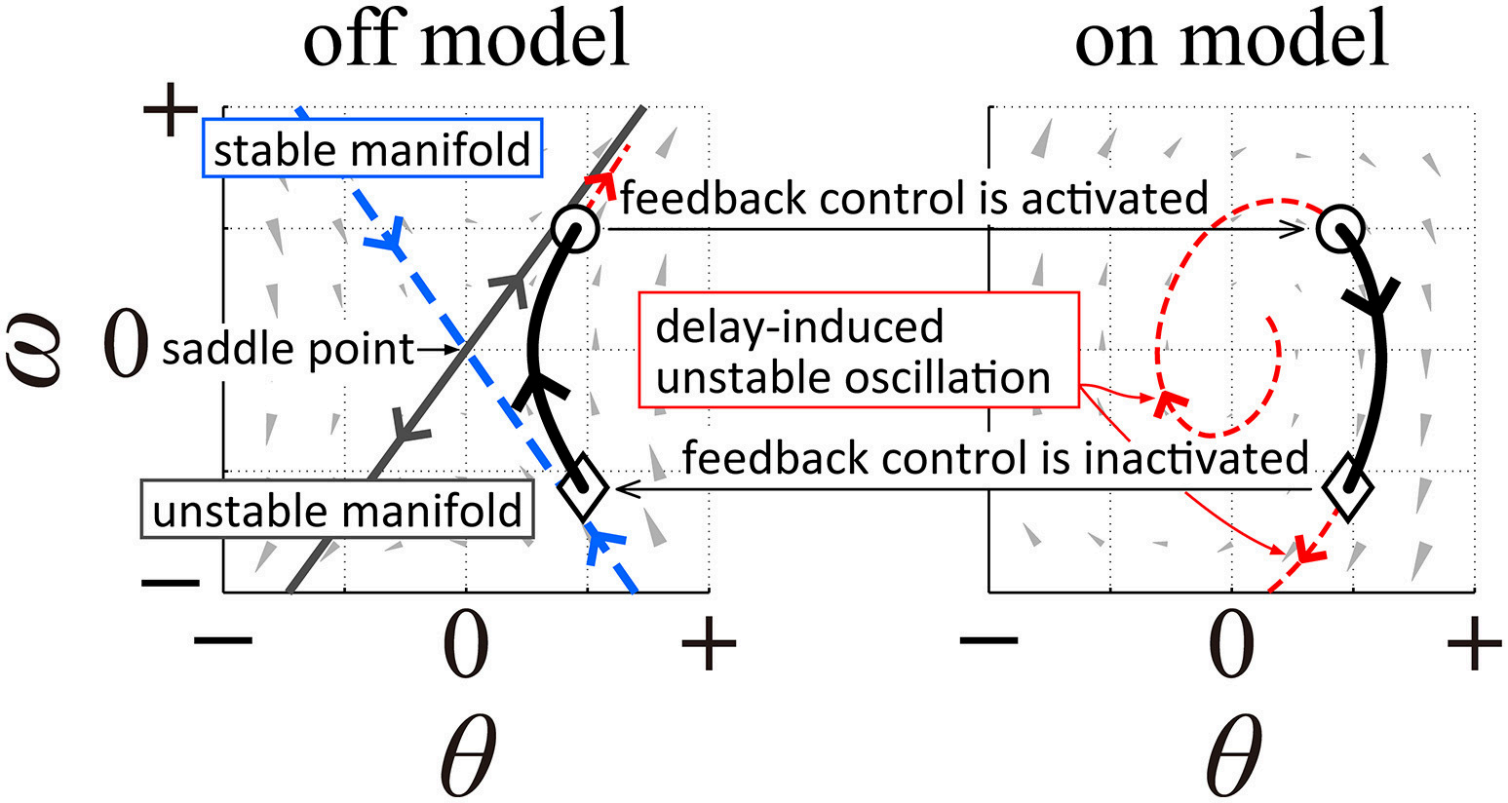
**A qualitative illustration of the intermittent feedback control model in human quiet stance based on a single inverted pendulum modeling as in Asai et al. (2009)**. The left panel illustrates typical dynamics of an inverted pendulum without active feedback control (off-model), where the upright posture (the origin of the phase plane) is a saddle-type unstable equilibrium point with stable and unstable manifolds. The right panel illustrates dynamics of the pendulum with time-delayed active feedback control (on-model), where the system shows unstable oscillation due to delay-induced instability. Appropriate state-dependent intermittent switching between these two unstable dynamics make the overall dynamics stable, characterized by a bounded stability (typically with limit cycle oscillation).

It is possible to associate the UCM, as well as postural variation along the UCM, with *the intermittent feedback control hypothesis* that has recently been proposed as a mechanism for stabilization during quiet standing, steady-state walking, and stick balancing (e.g., Bottaro et al., 2008; Asai et al., 2009; Suzuki et al., 2012; Fu et al., 2014; Yoshikawa et al., 2016). The intermittent feedback control hypothesis assumes intermittent inactivation of the active feedback controller, by which the CNS intermittently exploits purely mechanical, passive dynamics of the human body in the absence of active feedback control. More specifically, upright posture without active feedback control is a saddle-type, unstable equilibrium point (i.e., the saddle-point) in the state space (Bottaro et al., 2008; Asai et al., 2009, 2013; Suzuki et al., 2012; Morasso et al., 2014). Since the saddle-point is accompanied by both stable and unstable manifolds in the state space, the state point can transiently approach the saddle-point while exhibiting hyperbolic dynamics in the state space if it is close to the stable manifold. This would then be followed by a departure from the saddle-point along the unstable manifold and would determine a fall without feedback intervention. See Figure 1 (left panel) illustrating this situation, based on a single inverted pendulum modeling as in Asai et al. (2009). In this paper, the model of postural control without active feedback will be referred to as *the off-model*, describing purely mechanical, passive dynamics of the human body, while the system with active feedback, i.e., dynamics of the human body actuated by a neural feedback controller, will be referred to as *the on-model*. The intermittent feedback control model hypothesizes that the CNS alternates between the off- and on-models, and that this alternation depends on time delay-affected feedback information about the state point. That is, the intermittent controller prescribes that when the state point remains “near” the stable manifold of the off-model, the active feedback control is inactivated (switched off), thus allowing the state point to transiently approach the saddle-point without active feedback control. In contrast, when the state point shifts “far” away from the stable manifold, feedback control is re-activated (switching from the off-model to the on-model). Typically, the intermittent feedback control model operates with small feedback gains for the on-model (Asai et al., 2009; Suzuki et al., 2012), namely gain values that would be incapable to achieve stability even if feedback control were to remain persistently activated. Moreover, regardless of the feedback gain values, persistent feedback control is highly prone to delay-induced instability, leading to an expanding, divergent oscillation around the upright equilibrium point. On the other hand, such unstable dynamics can also be exploited as a kind of “affordance” because they provide an opportunity for the state point (or the sway trajectory) of the on-model to transverse the stable manifold of the off-model at some time during the globally unstable oscillation; inactivating the active feedback controller at that time (switching from the on-model to the off-model) would trigger another transient dynamic approach to the saddle-point along the stable manifold. See Figure 1 (right panel) illustrating this situation, based on a single inverted pendulum modeling as in Asai et al. (2009). What is remarkable is that although the two control models (off-model and on-model) are both unstable, the combination of the two, according to the switching mechanism described above, can achieve “bounded stability” (Bottaro et al., 2008) by constraining the position of the state point to a kind of limit cycle around the unstable upright equilibrium point. Another remarkable characteristic of the intermittent feedback control model is the small joint impedance that originates from the null feedback gain in the off-model and the small feedback gain in the on-model, leading to joint flexibility accompanied by moderate movement variability (Nomura et al., 2013). See Figure 1, in which state-dependent switching between the left and right panels would lead to a cyclic trajectory (a limit cycle) representing a postural sway during quiet standing without motor noise.

Interestingly, the stable manifold of the saddle-point in the off-model can be considered as a type of uncontrolled manifold because the feedback control is inactivated when the state point is located near the stable manifold. In this paper, we refer to the stable manifold of the off-model as the dynamic uncontrolled manifold (*dynamic-UCM*) because it is determined by dynamic equations of motion of the off-model, whereby the majority of postural sway in the intermittent feedback control model appears as joint motion in/along the dynamic-UCM. For double inverted pendulum modeling with typical body parameter values, the dynamic-UCM is defined as a three-dimensional stable manifold (the direct sum of a two- and one-dimensional stable manifold) in the four-dimensional state space of the off-model (Suzuki et al., 2012). The complemental subspace of the dynamic-UCM is a one-dimensional unstable manifold. Dynamic modes associated with the one-dimensional stable manifold and the one-dimensional unstable manifold in the off-model exhibit monotonic recovering and falling toward and from the vertical upright equilibrium, respectively, where ankle and hip joint angles both rotate in the same direction (*stable* and *unstable in-phase modes*, respectively). A dynamic mode associated with the two-dimensional stable manifold in the off-model exhibits a quasi-stable oscillation, where ankle and hip joint angles exhibit an anti-phase, damped oscillation (two joint angles alternating in opposite directions) toward upright equilibrium (*stable anti-phase mode*). Despite the fact that the equilibrium point of the on-model is unstable due to delay-induced instability (Suzuki et al., 2011), the upright posture of the double inverted pendulum model can be robustly stabilized by the intermittent feedback control strategy that exploits transiently convergent dynamics along the dynamic-UCM, particularly the two-dimensional stable manifold of the off-model (Suzuki et al., 2012). Thus, for the intermittent feedback control model with the double inverted pendulum, postural sway during off-phases appears as anti-phase coordinated movements between the ankle and hip joints near the two-dimensional stable manifold as a major part of the dynamic-UCM.

In this way, although mathematical definitions of the kinematic- and dynamic-UCMs are completely different, these two types of UCM play similar roles in the sense that the goal of the active feedback controller is to drive the postural state close to the UCM (either kinematic- or dynamic-UCM), while the CNS can suspend active interventions in postural control when the postural state is located near the UCM because the resulting intrinsic dynamics is indeed consistent with global stabilization. In this paper, we assimilated experimental sway data to the double inverted pendulum model, and analyzed kinematic- and dynamic-UCMs. We showed that geometric arrangements of the kinematic-UCM and two-dimensional stable manifolds of the dynamic-UCM in the joint angle and angular velocity spaces were too similar to be distinguished (at least with the resolution provided by postural sway measurements). Thus, we were unable to experimentally determine which hypothetical control strategy (i.e., CoM-control hypothesis or intermittent control hypothesis) was employed by the CNS to stabilize upright posture.

We then quantitatively investigated whether postural sway near the dynamic-UCM could be considered as purely mechanical, passive dynamics of the human body without active neural feedback control (i.e., off-model). In order to evaluate this question, we analyzed experimental postural sway data and dynamics of the off-model using the double inverted pendulum. It was expected that postural sway dynamics near the UCM could be characterized by dominant eigenvalues (eigenfrequency) associated with the dynamic-UCM, in which the uncontrolled body (off-model) would exhibit anti-phase coordinated oscillations of the ankle and hip joints (stable mode of unstable dynamics). We will show that this expectation is valid under certain conditions of passive joint impedance (passive viscoelasticity of the ankle and hip joints) that determine the eigenfrequency of the off-model in the dynamic-UCM. This is because large (small) passive joint elasticity likely results in high (low) eigenfrequency in the off-model. Thus, the expectation is that if the experimental postural sway near the kinematic-UCM were to exhibit high-frequency oscillations close to the eigenfrequency of the dynamic-UCM for a range of physiologically plausible passive joint impedances, we conclude that purely mechanical, passive dynamics (dynamics of the off-model) is responsible for postural sway near the UCM. At the same time, this would enable us to identify the passive joint-viscoelasticity of the ankle and hip by examining a range of elastic and viscous coefficients of the ankle and hip joints that are capable of producing a characteristic sway frequency close to the eigenfrequency of the dynamic-UCM.

## Materials and methods

**Figure 2:**
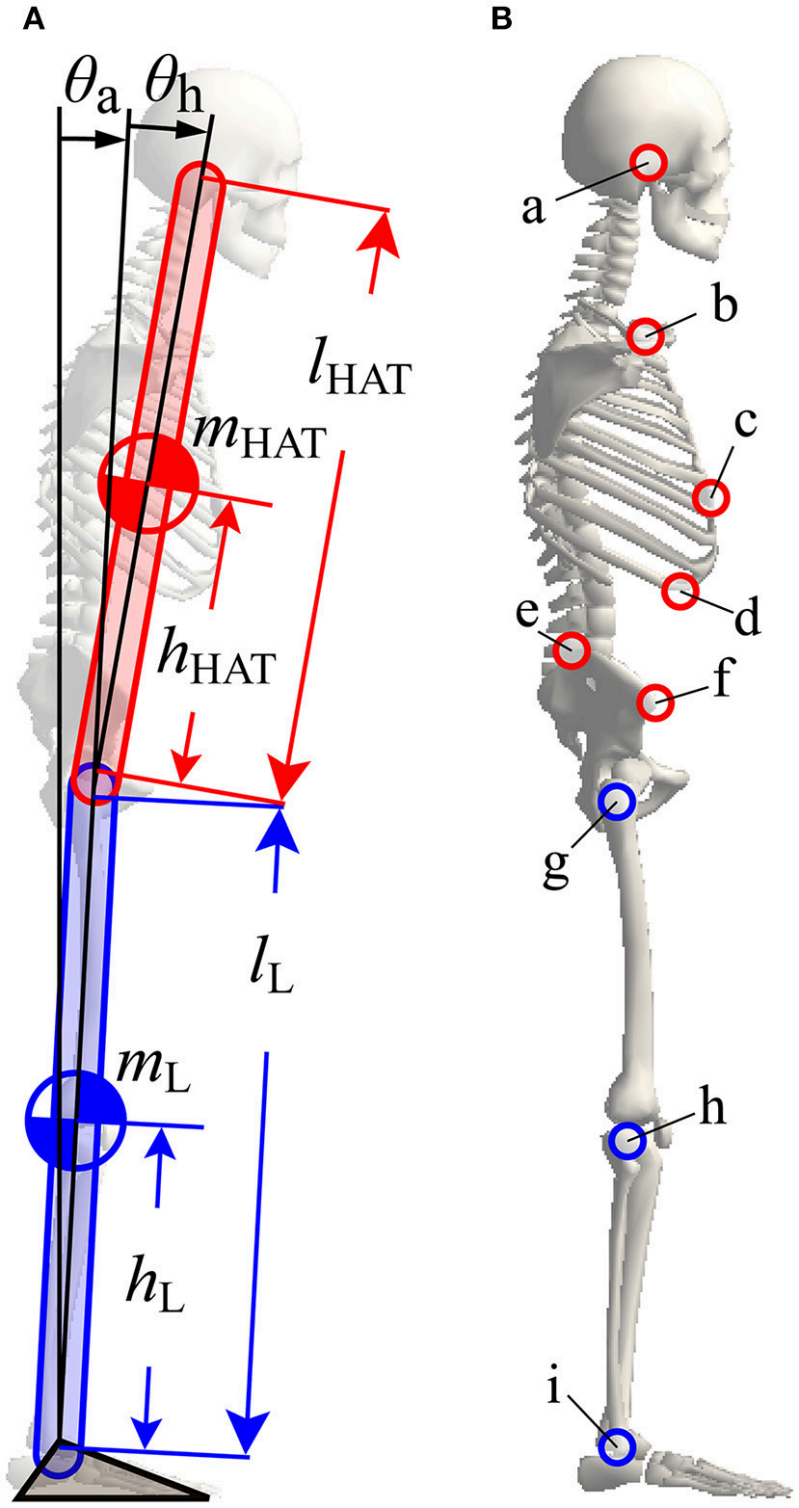
**A double inverted pendulum model of upright posture with marker positions used for the experimental measurement of posture during quiet stance. (A)** A double inverted pendulum model consisting of upper (HAT) and lower (L) links with ankle and hip joints. See Table 1 for symbols. **(B)** Positions of markers used for experimental measurements. All markers, except marker “c”, were mounted on left and right sides at the following anatomical landmarks, a, ear; b, shoulder; c, xiphoid; d, lower rib; e, iliac crest; f, anterior superior iliac spine; g, hip; h, knee; I, ankle. See Table 3 for details of marker names and positions.

In order to model human quiet stance, we considered a double inverted pendulum model working in the sagittal plane (Figure 2A), where *m*_*i*_ (*i* = {L, HAT}) and *l*_*i*_ are the mass and lengths of the lower (L) and upper (HAT) links, *h*_L_ is the distance from the ankle joint to the CoM of the lower link, and *h*_HAT_ is the distance from the hip joint to the CoM of the upper link (see also Table 1). θ_a_ and θ_h_ represent, respectively, the tilt angle of the lower link from the upright position, referred to simply as the ankle joint angle, and the hip joint angle defined as the angle between the upper and the lower links. Angular velocities of the ankle and hip joints are denoted as ω_*j*_ = *d*θ_*j*_/*dt* (*j* = {a, h}). Because the angular displacements and velocities of the ankle and hip joints are small during quiet stance, the following approximations were performed:

(1) $$\begin{array}{l} \sin\theta_{j}\approx \theta_{j},\cos\theta_{j}\approx 1,\sin\omega_{j}\approx \omega_{j},\cos\omega_{j}\approx 1. \end{array}$$

**Table 1:** **Variables and parameters for the double pendulum model**.

**Symbol**	**Description**	**Value/Unit**
θ_a_	Ankle joint angle	— rad
θ_h_	Hip joint angle	— rad
ω_a_	Ankle angular velocity	— rad/s
ω_h_	Hip angular velocity	— rad/s
	Total body mass, obtained from experimental data	— kg
*g*	Gravitational acceleration	9.8 m/s^2^
*m*_L_	Lower link mass	
*m*_HAT_	Upper link mass	
*l*_L_	Lower link length	— m
*l*_HAT_	Upper link length	— m
*h*_L_	Distance from the distal end to the CoM of the lower link	*l*_L_ × 0.5
*h*_HAT_	Distance from the distal end to the CoM of the upper link	*l*_HAT_ × 0.5
*m*	Total mass of the lower and upper links	*m*_L_ + *m*_HAT_
*h*	Height of the total CoM for θ_h_ = 0 (see Equation A.9)	— m
*K*_a_	Passive elastic coefficient at the ankle	0.8*mgh* Nm/rad
*B*_a_	Passive viscosity coefficient at the ankle joint	4.0 Nms/rad
*K*_h_	Representative passive elastic coefficient at the hip joint	0.4*mgh* Nm/rad
*B*_h_	Representative passive viscosity coefficient at the hip joint	10.0 Nms/rad
*P*_a_	Proportional gain of neural feedback control at the ankle	— Nm/rad
*D*_a_	Derivative gain of neural feedback control at the ankle	— Nms/rad
*P*_h_	Proportional gain of neural feedback control at the hip	— Nm/rad
*D*_h_	Derivative gain of neural feedback control at the hip	— Nms/rad

Thus, the linearized equation of motion for the double inverted pendulum model could be described as:

(2) $$\begin{array}{l} \text{M}\frac{d^{2}}{dt^{2}}\theta +\text{G}\theta =\text{Q}\equiv {\text{Q}}_{\text{p}}+{\text{Q}}_{\text{a}}, \end{array}$$

where **θ**=(θ_a_, θ_h_)^T^ is the joint angle vector, **M** the inertia matrix, and **Gθ** the gravitational toppling torque vector. **Q** represents the joint toque vector exerted at the ankle and hip joints, which is modeled by a sum of the passive torque vector (**Q**_p_) and the active torque vector (**Q**_a_). These vectors and matrices are defined in Appendix A (Supplementary Material). Note that the passive torque vector continuously acts on the ankle and hip joints without time-delay, since passive joint torques are generated by intrinsic mechanical properties of muscles, tendons, and soft tissues around the joints. On the other hand, active joint torques generated by the CNS are affected by the feedback time-delay due to neural signal processing and signal transmission.

### The kinematic-UCM

The anterior-posterior position of the CoM of the double inverted pendulum model (*x*_CoM_) was calculated as follows:

(3) $$\begin{array}{l} x_{\text{CoM}}=\frac{m_{\text{L}}h_{\text{L}}\theta_{\text{a}}+m_{\text{HAT}}\left\{l_{\text{L}}\theta_{a}+h_{\text{HAT}}\left(\theta_{\text{a}}+\theta_{\text{h}}\right)\right\}}{m_{\text{L}}+m_{\text{HAT}}}. \end{array}$$

The kinematic-UCM used for the CoM-control hypothesis is a low-dimensional space that satisfies a kinematic constraint such that the anterior-posterior position of the CoM does not change in time, i.e., *x*_CoM_ = constant (hence *dx*_CoM_/*dt* = 0). In the double inverted pendulum model, the kinematic-UCM is defined as the one-dimensional subspace in the joint angle space (θ_a_-θ_h_ plane) and angular velocity space (ω_a_-ω_h_ plane):

(4) $$\begin{array}{l} \theta_{\text{h}}=-\frac{m_{\text{L}}h_{\text{L}}+m_{\text{HAT}}l_{\text{L}}+m_{\text{HAT}}h_{\text{HAT}}}{m_{\text{HAT}}h_{\text{HAT}}}\theta_{\text{a}}+\text{constant}, \end{array}$$

(5) $$\begin{array}{l} \omega_{\text{h}}=-\frac{m_{\text{L}}h_{\text{L}}+m_{\text{HAT}}l_{\text{L}}+m_{\text{HAT}}h_{\text{HAT}}}{m_{\text{HAT}}h_{\text{HAT}}}\omega_{\text{a}}. \end{array}$$

In this study, we set the constant in Equation (4) as **0** for simplicity, i.e., we assumed a vertically upright posture as the equilibrium of the system. From Equations (4) and (5), the kinematic-UCM forms a straight line with a negative slope, passing through the origin both in the θ_a_-θ_h_ and ω_a_-ω_h_ planes. Note that negative slopes in the θ_a_-θ_h_ and ω_a_-ω_h_ planes are determined only by body parameters, and are exactly the same. In this way, the kinematic-UCM is defined only by the static balance of the body in the gravitational force field, and independent of the equation of motion (i.e., body dynamics).

### The off-model of the intermittent control system and the dynamic-UCM

For the off-model, the joint torque vector includes only the passive torque vector **Q**_p_, i.e., **Q**_a_ = **0** and **Q** = **Q**_p_, since the active neural feedback control is inactivated in the off-model. In this study, we modeled the passive torque using a linear viscoelastic element as follows:

(6) $$\begin{array}{l} {\text{Q}}_{\text{p}}={\text{T}}_{\text{p}}\left(\begin{array}{c} \theta_{\text{a}}\\ \theta_{\text{h}}\\ \omega_{\text{a}}\\ \omega_{\text{h}} \end{array}\right), \end{array}$$

where the matrix **T**_p_ represents the viscoelasticity matrix defined in Equation (A.5) of Appendix A (Supplementary Material), determined by the elastic and the viscous coefficients of the ankle and hip joints (*K*_a_, *B*_a_, *K*_h_, *B*_h_). Then, the state space representation of the off-model in the four-dimensional state space (θ_a_-θ_h_-ω_a_-ω_h_ space) is obtained as follows:

(7) $$\begin{array}{l} \frac{d}{dt}\left(\begin{array}{c} \theta_{\text{a}}\\ \theta_{\text{h}}\\ \omega_{\text{a}}\\ \omega_{\text{h}} \end{array}\right)=\left\{\left(\begin{array}{cc} 0 & \text{I}\\ -{\text{M}}^{-1}\text{G} & 0 \end{array}\right)+\left(\begin{array}{c} 0\\ {\text{M}}^{-1}{\text{T}}_{\text{p}} \end{array}\right)\right\}\left(\begin{array}{c} \theta_{\text{a}}\\ \theta_{\text{h}}\\ \omega_{\text{a}}\\ \omega_{\text{h}} \end{array}\right)\equiv \text{A}\left(\begin{array}{c} \theta_{\text{a}}\\ \theta_{\text{h}}\\ \omega_{\text{a}}\\ \omega_{\text{h}} \end{array}\right), \end{array}$$

where **I** is the 2 × 2 identity matrix, and **A** is the system matrix of the off-model. The four eigenvalues of matrix **A** of the off-model can be characterized as follows: a positive real pole, a negative real pole, and a pair of complex conjugate poles with a negative real part, for typical link parameters as those used by Suzuki et al. (2012). Thus, the upright posture of the off-model is a saddle-type unstable equilibrium point, accompanied by a one-dimensional unstable manifold and a three-dimensional stable manifold. According to Suzuki et al. (2012), the stable manifold can be decomposed into a one- and two-dimensional stable manifolds with the latter corresponding to the oscillatory anti-phase mode in the state space. On the other hand, the unstable and stable one-dimensional manifolds correspond to the unstable and stable in-phase modes, respectively. For the intermittent control hypothesis, the dynamic-UCM is defined as the three-dimensional stable manifold consisting of both one- and two-dimensional stable manifolds of the off-model. In this paper, we refer to the one-dimensional stable manifold as the D-UCM_in_ and the two-dimensional stable manifold as the D-UCM_anti_. Moreover, since the two-dimensional stable manifold has a prominent role in the intermittent feedback control model (Suzuki et al., 2012), this study focused mainly on the D-UCM_anti_. Table 2 summarizes the definition of the dynamic UCM.

**Table 2:** **Definition of the dynamic-UCM**.

System matrix of the off-model (4-dimensional)	Positive real pole	Unstable in-phase mode	
	Negative real pole	Stable in-phase mode	D-UCM_in_
	Pair of complex conj. poles with negative real part	Stable anti-phase mode	D-UCM_anti_

### The on-model of the intermittent control system

The on-model with the active joint torques at the ankle and hip joints was defined as follows:

(8) $$\begin{array}{l} \frac{d}{dt}\left(\begin{array}{c} \theta_{\text{a}}\\ \theta_{\text{h}}\\ \omega_{\text{a}}\\ \omega_{\text{h}} \end{array}\right)=\text{A}\left(\begin{array}{c} \theta_{\text{a}}\\ \theta_{\text{h}}\\ \omega_{\text{a}}\\ \omega_{\text{h}} \end{array}\right)+\left(\begin{array}{c} 0\\ {\text{M}}^{-1}{\text{T}}_{\text{a}} \end{array}\right)\left(\begin{array}{c} \theta_{\text{a}\Delta }\\ \theta_{\text{h}\Delta }\\ \omega_{\text{a}\Delta }\\ \omega_{\text{h}\Delta } \end{array}\right)\\ \equiv \;\text{A}\left(\begin{array}{c} \theta_{\text{a}}\\ \theta_{\text{h}}\\ \omega_{\text{a}}\\ \omega_{\text{h}} \end{array}\right)+{\text{A}}_{\text{a}}\left(\begin{array}{c} \theta_{\text{a}\Delta }\\ \theta_{\text{h}\Delta }\\ \omega_{\text{a}\Delta }\\ \omega_{\text{h}\Delta } \end{array}\right) \end{array}$$

where θ_*aΔ*_ ≡ θ_a_(*t* − Δ), θ_hΔ_ ≡ θ_h_(*t* − Δ), ω_aΔ_ ≡ ω_a_(*t* − Δ), and ω_hΔ_ ≡ ω_h_(*t* − Δ) are the state variables affected by the feedback time-delay Δ. The generation of active control torques is provided by a proportional and derivative (PD) feedback controller with time-delay: **T**_**a**_ is the feedback gain matrix with a set of proportional (*P*) and derivative (*D*) gains, i.e., *P*_a_ and *D*_a_ for the ankle joint, and *P*_h_ and *D*_h_ for the hip joint. See Equation (A.5) of Appendix A (Supplementary Material) for details. The equilibrium point of the on-model serves as the origin (θ_a_, θ_h_, ω_a_, ω_h_) = (0, 0, 0, 0), which is stable only for very narrow parameter regions due to delay-induced stability: see Suzuki et al. (2012) for details of how stability of the equilibrium point and stability regions of the on-model in the *P*_a_-*D*_a_-*P*_h_-*D*_h_ parameter space are determined.

### Experimental sway measurement

Five healthy young men (all 23-years-old, means ± SD: height 1.72 ± 0.05 m, weight 66.6 ± 12.72 kg) participated in this study. All subjects provided written informed consent for the study. The experimental procedures were in accordance with the Declaration of Helsinki, and they were approved by the ethical committee for human studies at the Graduate School of Engineering Science, Osaka University. This study did not employ female and elderly subjects. The investigation of a possible gender-bias and age-bias is left to a future study.

Subjects were asked to quietly stand barefoot on a platform with their arms hanging beside their body and their feet along a V-shaped guide, which was an isosceles triangle with a 20-degree vertex angle that separated the two malleoli by about 2 cm. Subjects were required to gaze at a fixation point that was displayed at eye-level height, about 2 m away from them. Five trials of quiet standing were performed—each lasting 70 s—with a sufficient resting period between the trials.

Seventeen infrared reflective markers were attached to the characteristic points on the body of each subject (details are shown in Figure 2B and Table 3). Three-dimensional positions of these markers were captured using a motion capture system (SMART-DX, BTC, Milan), which consisted of six infrared cameras, with a sampling frequency of 300 Hz. Captured marker positions were projected onto the sagittal plane, where the *x*- and the *y*-coordinates represented the anterior-posterior and superior-inferior directions of a subject's body, respectively.

**Table 3:** **List of markers for motion capturing: symbols and positions of markers that were used for identifying posture**.

**Body segment**	**Marker symbol**	**Description of marker position**
**HEAD ARM TRUNK**		
Head	R-Ear	Right tragion
	L-Ear	Left tragion
Trunk 4 and Arms	R-Shoulder	Right acromion
	L-Shoulder	Left acromion
	Xiphoid	Inferior end of the xiphoid
Trunk 3	Xiphoid	Inferior end of the xiphoid
	R-LowerRib	Right inferior thoracic aperture
	L-LowerRib	Left inferior thoracic aperture
Trunk 2	R-LowerRib	Right inferior thoracic aperture
	L-LowerRib	Left inferior thoracic aperture
	R-IliacCrest	Right superior end of the Iliac crest
	L-IliacCrest	Left superior end of the Iliac crest
Trunk 1	R-IliacCrest	Right superior end of the Iliac crest
	L-IliacCrest	Left superior end of the Iliac crest
	R-ASIS	Right anterior superior iliac spine
	L-ASIS	Left anterior superior iliac spine
Pelvis	R-ASIS	Right anterior superior iliac spine
	L-ASIS	Left anterior superior iliac spine
**LOWER LIMB**		
Thigh	R-Hip	Right greater trochanter
	L-Hip	Left greater trochanter
	R-Knee	Right lateral epicondyle
	L-Knee	Left lateral epicondyle
Lower Leg	R-Knee	Right lateral epicondyle
	L-Knee	Left lateral epicondyle
	R-Ankle	Right lateral malleolus
	L-Ankle	Left lateral malleolus

Time series data of the marker positions were digitally processed offline using a fourth-ordered Butterworth filter with zero phase lag and a cutoff frequency of 10 Hz. Using the filtered marker data, we estimated the lengths of *l*_L_ and *l*_HAT_ and the time-series of the tilt angles of the lower link φ_L_[*n*] and the HAT-link φ_HAT_[*n*] from the vertical direction, where *n* represents the discretized (sampling) time. See Appendix B (Supplementary Material) for details of the estimation method. Then, the ankle and hip joint angles at time *n*, θ_a_[*n*] and θ_h_[*n*], and their angular velocities, ω_a_[*n*] and ω_h_[*n*], were obtained as follows:

(9) $$\begin{array}{l} \theta_{a}\left[n\right]=\varphi_{\text{L}}\left[n\right]-\frac{1}{N}\overset{N}{\underset{n=1}{\sum }}\varphi_{\text{L}}\left[n\right], \end{array}$$

(10) $$\begin{array}{l} \theta_{\text{h}}\left[n\right]=\varphi_{\text{HAT}}\left[n\right]-\varphi_{\text{L}}\left[n\right]-\frac{1}{N}\overset{N}{\underset{n=1}{\sum }}\left(\varphi_{\text{HAT}}\left[n\right]-\varphi_{\text{L}}\left[n\right]\right), \end{array}$$

(11) $$\begin{array}{l} \omega_{\text{a}}\left[n\right]=\frac{\theta_{\text{a}}\left[n+1\right]-\theta_{\text{a}}\left[n-1\right]}{2\Delta t}, \end{array}$$

(12) $$\begin{array}{l} \omega_{\text{h}}\left[n\right]=\frac{\theta_{\text{h}}\left[n+1\right]-\theta_{\text{h}}\left[n-1\right]}{2\Delta t}, \end{array}$$

where Δ*t* is the sampling interval (1/300 s), and *N* is the total data number. In Equations (9) and (10), the mean values of φ_L_[*n*] and φ_HAT_[*n*]−φ_L_[*n*] are subtracted, respectively, in order to set the mean values of θ_a_[*n*] and θ_h_[*n*] as zeros so that the CoM of the vertically upright posture is zero.

### Visualization of the kinematic- and dynamic-UCMs

The double inverted pendulum model with parameter values (*l*_L_, *l*_HAT_, *h*_L_, *h*_HAT_, *m*_L_, and *m*_HAT_) for analyzing the postural sway of each subject was prepared, where the lengths (*h*_L_ and *h*_HAT_) and masses (*m*_L_ and *m*_HAT_) of the two links were estimated using the weight of each subject and his lower extremity to upper body ratio (see Winter et al., 1998); Table 1. We then visualized the kinematic- and dynamic-UCMs (D-UCM_in_ and D-UCM_anti_) of the double inverted pendulum model for each subject in the θ_a_-θ_h_ and ω_a_-ω_h_ planes. The kinematic-UCM could be determined only by the kinematic body parameters of Equations (4) and (5), and was easily depicted as a straight line in each plane. However, visualization of the dynamic-UCM required specific values of passive viscoelasticity for the ankle and hip joints. In this study, the passive elastic and viscosity coefficients of the ankle joint were set as (*K*_a_, *B*_a_) = (0.8*mgh*, 4.0 Nms/rad) as in Table 1 (Loram and Lakie, 2002; Casadio et al., 2005). Since no experimental evaluation of the passive viscoelasticity for the hip joint during quiet stance has been published, we considered a set of small hip joint values as (*K*_h_, *B*_h_) = (0.4*mgh*, 10.0 Nms/rad) which were based on the idea that the passive impedance of the hip joint would be small, as in the ankle joint (see Table 1). However, since dynamics of the double inverted pendulum could be affected by the passive viscoelasticity of the hip joint, in the last half of this study, we analyzed these dynamics using various values of the passive viscoelasticity of the hip joint.

**Figure 3:**
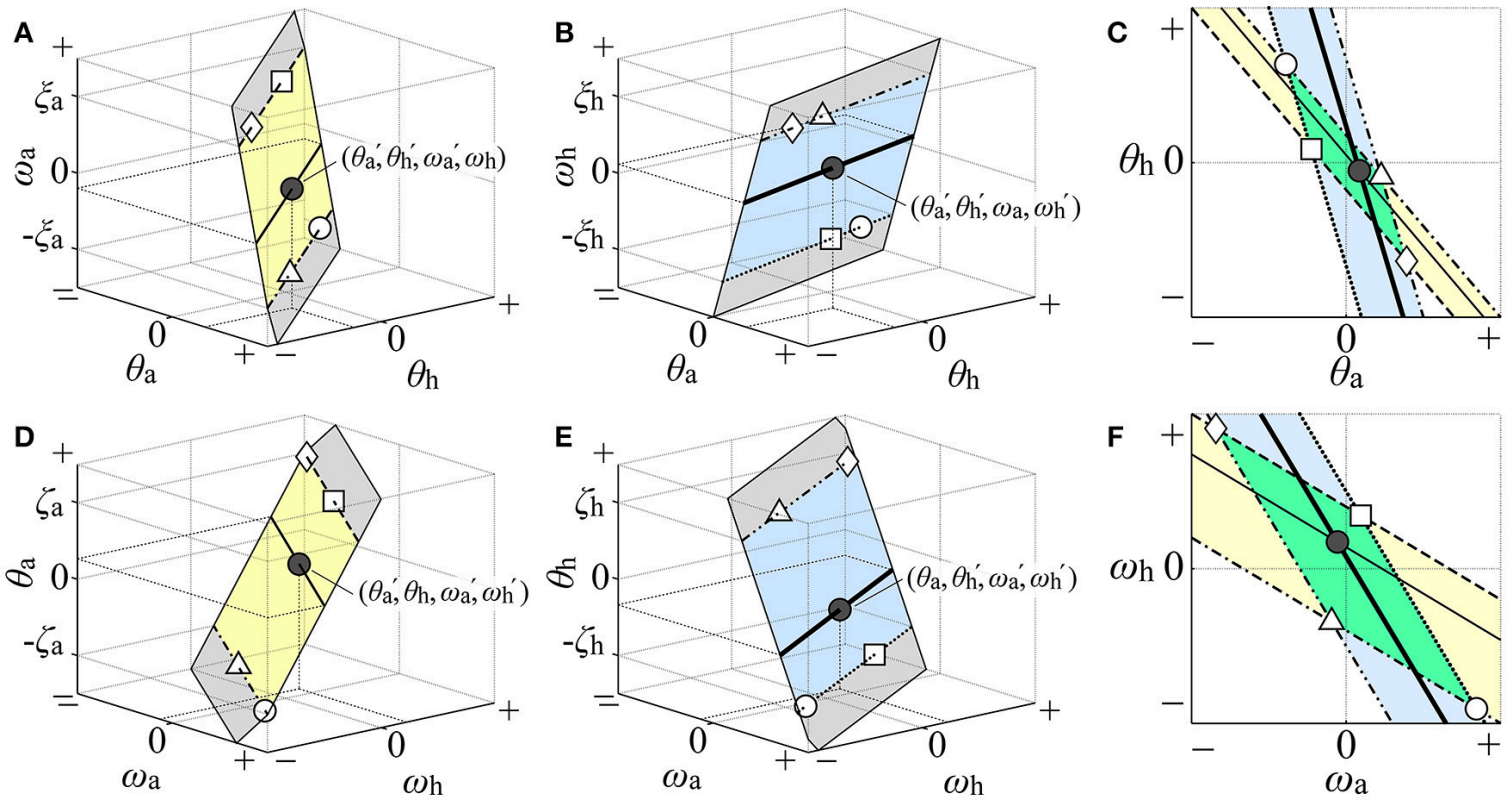
**Illustration of how the two-dimensional dynamic-UCM (D-UCM_**anti**_) in the four-dimensional state space was visualized in the θ_**a**_-θ_**h**_ and ω_**a**_-ω_**h**_ planes**. The gray planes in **(A,B,D,E)** illustrate the D-UCM_anti_ qualitatively in the θ_a_-θ_h_-ω_a_, θ_a_-θ_h_-ω_h_, ω_a_-ω_h_-θ_a_, and ω_a_-ω_h_-θ_h_ spaces, respectively. Restricted area of the D-UCM_anti_ in each of θ_a_-θ_h_-ω_a_ space [the yellow band of infinite length in **(A)**] and the θ_a_-θ_h_-ω_h_ space [the blue band of infinite length in **(B)**] such that each area comparable with the range of postural sway during quiet stance is projected on the θ_a_-θ_h_ plane in the **(C)**. Geometrical configuration of the D-UCM_anti_ in the θ_a_-θ_h_ plane, visually characterized by the parallelogram formed by overlapping regions of two slanted bands in the θ_a_-θ_h_ plane of the **(C)**. Similarly, the restricted area of the D-UCM_anti_ in the ω_a_-ω_h_-θ_a_ space [yellow band in **(D)**] and ω_a_-ω_h_-θ_h_ space [blue band in **(E)**] is projected on the ω_a_-ω_h_ plane in the **(F)**. Geometrical configuration of the D-UCM_anti_ in the ω_a_-ω_h_ plane, visually characterized by the parallelogram formed by the overlapping regions of the two slanted bands in the θ_a_-θ_h_ plane of **(F)**. See main text for details.

Rigorous visualization of the two-dimensional D-UCM_anti_ in the θ_a_-θ_h_ and ω_a_-ω_h_ planes is not easy because it is defined in the four-dimensional state space. Nevertheless, we tried to perform this visualization [detailed in Appendix C (Supplementary Material)] using four types of three-dimensional space: θ_a_-θ_h_-ω_a_ (Figure 3A), θ_a_-θ_h_-ω_h_ (Figure 3B), ω_a_-ω_h_-θ_a_ (Figure 3D), and ω_a_-ω_h_-θ_h_ (Figure 3E). Note that, in each panel of Figure 3, the D-UCM_anti_ is visualized by a two-dimensional plane for illustrative purposes. The two-dimensional D-UCM_anti_ planes in the θ_a_-θ_h_-ω_a_ (Figure 3A) and θ_a_-θ_h_ -ω_h_ (Figure 3B) spaces were projected on the single θ_a_-θ_h_ plane, as shown in Figure 3C, which could be compared with the kinematic-UCM in the θ_a_-θ_h_ plane defined by Equation (4). Similarly, the D-UCM_anti_ planes in the ω_a_-ω_h_-θ_a_ (Figure 3D) and ω_a_-ω_h_-θ_h_ (Figure 3E) spaces were projected on the ω_a_-ω_h_ plane, as in Figure 3F, which could be compared with the kinematic-UCM in the ω_a_-ω_h_ plane defined by Equation (5). In the result section, we will show that these two projections are almost identical with the kinematic-UCMs defined by Equations (4) and (5).

Since mapping of the whole two-dimensional D-UCM_anti_ space would inevitably cover the entire θ_a_-θ_h_ plane, we considered a restricted area of the D-UCM_anti_ in each of θ_a_-θ_h_-ω_a_ and θ_a_-θ_h_-ω_h_ space such that each area would be comparable with the range of postural sway during quiet stance: one area of the D-UCM_anti_ (yellow band of infinite length in Figure 3A) restricted by the upper bound of ω_a_ at ξ_a_ (dashed line in Figure 3A) and the lower bound of ω_a_ at −ξ_a_ (dot-dashed line in Figure 3A) in the θ_a_-θ_h_-ω_a_ space. The other area of the D-UCM_anti_ (blue band of infinite length in Figure 3B) was restricted by the upper bound of ω_h_ at ξ_h_ (double dot-dashed line in Figure 3B) and the lower bound of ω_h_ at –ξ_h_ (dotted line in Figure 3B) in the θ_a_-θ_h_-ω_h_ space. Each restricted area was then mapped onto the θ_a_-θ_h_ plane for visualization of the D-UCM_anti_ in the θ_a_-θ_h_ plane. To be specific, we considered a mapping of a four-dimensional state point ($\theta_{\text{a}}^{\prime }$, $\theta_{\text{h}}^{\prime }$, $\omega_{\text{a}}^{\prime }$, $\omega_{\text{h}}^{\prime }$) in the D-UCM_anti_ to the θ_a_-θ_h_ plane. This state point is represented by a point (θ_a_, θ_h_, ω_a_) = ($\theta_{\text{a}}^{\prime }$, $\theta_{\text{h}}^{\prime }$, $\omega_{\text{a}}^{\prime }$) in the yellow band, as indicated by the black point in Figure 3A. Since this point represents a set of state points (θ_a_, θ_h_, ω_a_, ω_h_) = ($\theta_{\text{a}}^{\prime }$, $\theta_{\text{h}}^{\prime }$, $\omega_{\text{a}}^{\prime }$, ω_h_) with an arbitrary value of ω_h_, it cannot uniquely specify a single state point. Moreover, it is not necessarily the state point ($\theta_{\text{a}}^{\prime }$, $\theta_{\text{h}}^{\prime }$, $\omega_{\text{a}}^{\prime }$, $\omega_{\text{h}}^{\prime }$) in the D-UCM_anti_. For specifying the four-dimensional state point ($\theta_{\text{a}}^{\prime }$, $\theta_{\text{h}}^{\prime }$, $\omega_{\text{a}}^{\prime }$, $\omega_{\text{h}}^{\prime }$) in the D-UCM_anti_, we need to specify one more point (θ_a_, θ_h_, ω_h_) = ($\theta_{\text{a}}^{\prime }$, $\theta_{\text{h}}^{\prime }$, $\omega_{\text{h}}^{\prime }$) in the blue band, as indicated by the black point in Figure 3B, which shares the same coordinate values (i.e., θ_a_ = $\theta_{\text{a}}^{\prime }$ and θ_h_ = $\theta_{\text{h}}^{\prime }$) as the black point in Figure 3A. However, by itself, this point represented a set of points (θ_a_, θ_h_, ω_a_, ω_h_) = ($\theta_{\text{a}}^{\prime }$, $\theta_{\text{h}}^{\prime }$, ω_a_, $\omega_{\text{h}}^{\prime }$) with an arbitrary value of ω_a_. In this way, the state point ($\theta_{\text{a}}^{\prime }$, $\theta_{\text{h}}^{\prime }$, $\omega_{\text{a}}^{\prime }$, $\omega_{\text{h}}^{\prime }$) in the D-UCM_anti_ is visualized using both Figures 3A,B, and is mapped (projected) to the black point (θ_a_, θ_h_) = ($\theta_{\text{a}}^{\prime }$, $\theta_{\text{h}}^{\prime }$) in the θ_a_-θ_h_ plane of Figure 3C. In the same way, the thin-solid line in the yellow band of the θ_a_-θ_h_-ω_a_ space and the thick-solid line in the blue band of the θ_a_-θ_h_-ω_h_ space are mapped to the corresponding lines, respectively, in the θ_a_-θ_h_ plane (Figure 3C). Similarly, the dashed and dot-dashed lines in the upper and lower bounds (ξ_a_, –ξ_a_) = (0.03, –0.03) of the yellow band in Figure 3A are mapped to the corresponding lines that forms the slanted yellow band in Figure 3C. Moreover, the dots-dashed and dotted lines of the upper and lower bounds (ξ_h_, –ξ_h_) = (0.03, –0.03) of the blue band in Figure 3B are mapped to the corresponding lines that forma the slanted blue band in Figure 3C. Since the angular velocities of the ankle and hip joints during quiet stance are mostly in the range of [−0.03, 0.03] rad/sec, we characterized the restricted area of the D-UCM_anti_ projected to the θ_a_-θ_h_ plane by a green parallelogram, which is formed by the intersection of the two slanted bands (Figure 3C). As the ankle and hip joint angles during quiet stance are mostly in the range of [−0.02, 0.02] rad, we visualized the geometry of D-UCM_anti_ in the ω_a_-ω_h_ plane in a similar manner (i.e., by a parallelogram formed by overlapping regions of the slanted yellow band (mapped from the yellow band in Figure 3D with upper and lower bounds at (ζ_a_, –ζ_a_) = (0.02, –0.02)) and slanted blue band (mapped from the blue band in Figure 2E with upper and lower bounds at (ζ_h_, –ζ_h_) = (0.02, –0.02)) as in Figure 3F.

### Comparison among postural sway dynamics; kinematic- and dynamic-UCMs

For sway data from each of the five experimental trials, angles and angular velocities of the ankle and hip joints were plotted as trajectories in the θ_a_-θ_h_ and ω_a_-ω_h_ planes, respectively, to examine whether they moved dominantly in the kinematic- and/or dynamic-UCM. In particular, we expected that high-frequency components of joint motion (i.e., above 1 Hz), which are known to exhibit anti-phase coordinated patterns between the ankle and hip joints (Creath et al., 2005; Zhang et al., 2007; Kato et al., 2014), would be constrained in the kinematic- and/or dynamic-UCM. To examine this expectation, experimental data related to the joint angles and angular velocities were low- and high-pass filtered using a fourth-order Butterworth filter with zero phase lag and a cut-off frequency of 1 Hz. The low-frequency (low-pass filtered data) and high-frequency (high-pass filtered data) components were also plotted separately in the θ_a_-θ_h_ and ω_a_-ω_h_ planes, respectively, to examine whether they moved in the kinematic- and/or dynamic-UCM.

The geometry of the kinematic-UCM in the θ_a_-θ_h_ and ω_a_-ω_h_ planes was characterized by the slope of the lines defined by Equations (4) and (5). The geometries of the D-UCM_anti_ and experimental sway trajectories in the θ_a_-θ_h_ and ω_a_-ω_h_ planes were characterized using the principal component analysis (PCA). For characterizing the D-UCM_anti_, PCA was applied to the coordinates of the four parallelogram vertices defined above. For each of the D-UCM_anti_ and sway trajectories, we calculated eigenvectors of the principal components in the θ_a_-θ_h_ and ω_a_-ω_h_ planes, denoted by ($\theta_{\text{a}}^{\text{p}}$, $\theta_{\text{h}}^{\text{p}}$)^T^ and ($\omega_{\text{a}}^{\text{p}}$, $\omega_{\text{h}}^{\text{p}}$)^T^, respectively, as well as the contribution rates of the first principal components. Geometries of the D-UCM_anti_ and experimental sway trajectories were characterized by the slopes of the first principal eigenvectors (γ_θ_ and γ_ω_), which are defined as follows:

(13) $$\begin{array}{ll} \gamma_{\theta }=\frac{\theta_{\text{h}}^{\text{p}}}{\theta_{\text{a}}^{\text{p}}}, & \gamma_{\omega }=\frac{\omega_{\text{h}}^{\text{p}}}{\omega_{\text{a}}^{\text{p}}}. \end{array}$$

### Spectral characterization of anti-phase coordinated sway

The high-frequency component (>1 Hz) of joint motion during quiet stance, which was expected to move along the kinematic-UCM (and the D-UCM_anti_, as revealed in this study), was quantitatively analyzed in order to examine whether the postural sway near the kinematic-UCM (and the dynamic-UCM) was generated by purely mechanical, passive dynamics of the human body. In other words, having no active neural feedback control (the off-model). To this end, we performed a spectral analysis by estimating power spectral density (PSD) functions of the experimental joint angles and angular velocities of each joint. Specifically, the time-series data were divided into segments of 10 s with 50% overlap, and the linear trend in each segment was removed. Then, a fast Fourier transform with Blackman-Harris window was applied to each segment, and an ensemble average of the spectra for all segments of the five trials was performed to estimate the PSD for each subject.

### Parameter-dependence of the dynamic-UCM eigenfrequency

Characteristic peak frequencies of the PSD were then compared with the eigenfrequency of the anti-phase mode (D-UCM_anti_) of the off-model in the double inverted pendulum model. The eigenfrequency of the D-UCM_anti_ was calculated from an imaginary part of the complex eigenvalue associated with the eigenvector that spanned the D-UCM_anti_. Since the eigenfrequency depends on the passive viscoelasticity of each joint, we calculated the eigenfrequency of the anti-phase mode of the off-model by varying the elastic and viscosity coefficients of the hip joint over a wide range, as follows:

(14) $$K_{\text{h}}\in \left[\begin{array}{cc} 0.2mgh & 1.0mgh \end{array}\right],B_{\text{h}}\in \left[\begin{array}{cc} 0 & 50 \end{array}\right]$$

where *m* and *h* are the total body mass and the distance between ankle and the total CoM, respectively. *g* represents the gravitational constant. According to most previous studies, and based on experimental measurements, the passive elastic and viscosity coefficients of the ankle joint were fixed as (*K*_a_, *B*_a_) = (0.8*mgh*, 4.0) (Table 1, Maurer and Peterka, 2005; Bottaro et al., 2008; Asai et al., 2009; Suzuki et al., 2012).

Although it has been shown that the ankle and hip joints move around the kinematic-UCM in a coordinated manner, no quantitative examinations have evaluated whether coordinated joint motion is really a consequence of “uncontrolled” body dynamics (i.e., the off-model) without active neural feedback control. If this were the case, then the PSD of the high-frequency component of joint motion (expected to move along the kinematic-UCM) would exhibit characteristic peaks that are coincident with the eigenfrequency of the anti-phase mode of the off-model. On the other hand, if the characteristic frequencies of the PSD were to coincide with the eigenfrequency of the anti-phase mode of the on-model (i.e., if the postural sway near the kinematic-UCM could be better explained by dynamics with active neural feedback control), then the UCM hypotheses (both the CoM-control hypothesis and intermittent feedback control hypothesis) might not be good candidate control strategies exploited by the CNS during quiet stance. In order to explore these possibilities, we analyzed eigenfrequency of the anti-phase mode and upright equilibrium stability of the on-model by systematically varying elastic and viscosity coefficients of the hip joint, as well as the proportional and derivative gains of the active feedback controller of the ankle and hip joints, over a wide range as follows [see Appendix D (Supplementary Material) for details]:

(15) $$\begin{array}{cc} K_{\text{h}}\in \left[\begin{array}{cc} 0.2mgh & 1.0mgh \end{array}\right], & B_{\text{h}}\in \left[\begin{array}{cc} 0 & 50 \end{array}\right],\\ P_{\text{a}}\in \left[\begin{array}{cc} 0.2mgh & 1.0mgh \end{array}\right], & D_{\text{a}}\in \left[\begin{array}{cc} 0 & 200 \end{array}\right],\\ P_{\text{h}}\in \left[\begin{array}{cc} 0.2mgh & 1.0mgh \end{array}\right], & D_{\text{h}}\in \left[\begin{array}{cc} 0 & 50 \end{array}\right]. \end{array}$$

## Results

**Figure 4:**
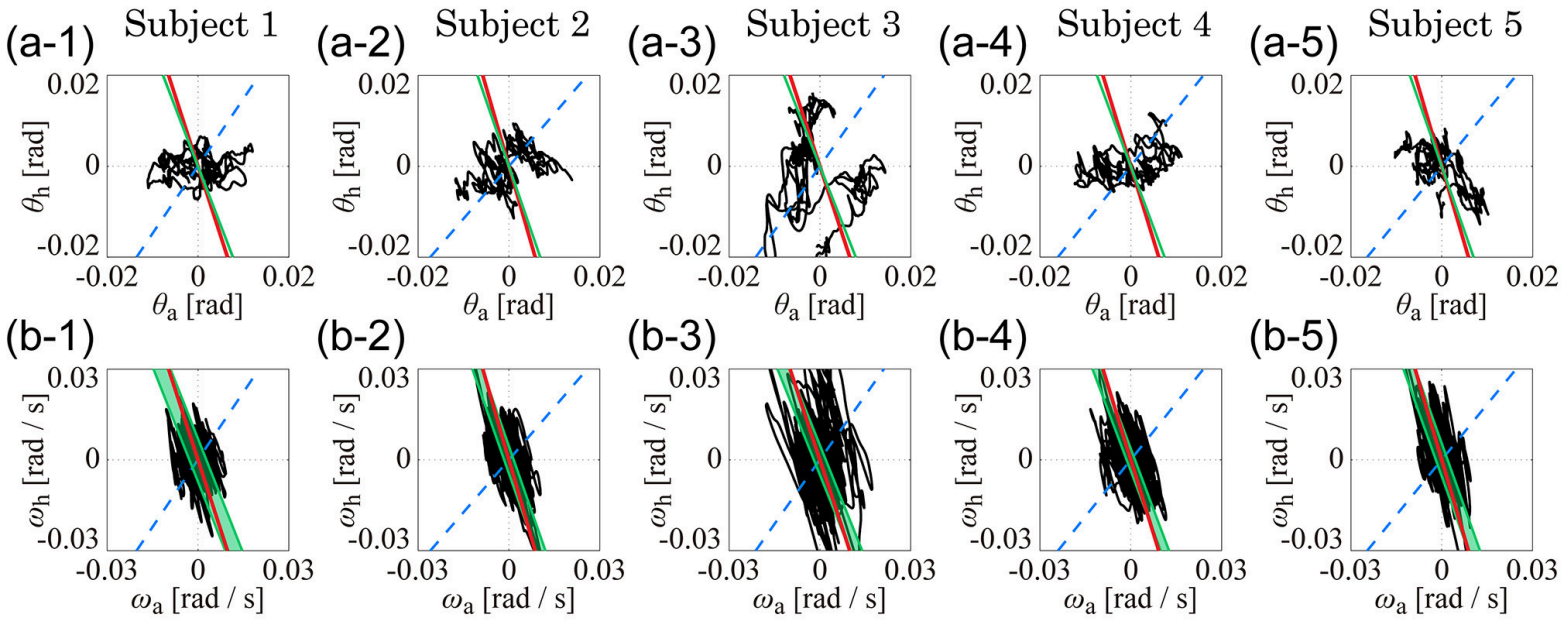
**The kinematic- and dynamic-UCMs (i.e., D-UCM_**in**_ and D-UCM_**anti**_, respectively) of the double inverted pendulum model, and sample trajectories of ankle and hip joint movement during quiet stance for five subjects**. In each panel, the red line represents the kinematic-UCM and the green band (which is a parallelogram defined in Figure 3) represents the D-UCM_anti_. The blue-dashed line represents the D-UCM_in_. The black curve is a sample trajectory of experimental postural sway. **(A1–A5)** are for the θ_a_-θ_h_ plane and **(B1–B5)** are for the ω_a_-ω_h_ plane.

Figure 4 shows the kinematic-UCM, dynamic-UCM (D-UCM_in_ and D-UCM_anti_), and experimental sway trajectories for the angles and angular velocities of the ankle and hip joints in the θ_a_-θ_h_ plane (Figures 4A1–A5) and in the ω_a_-ω_h_ plane (Figures 4B1–B5) for each of the five subjects. In each panel, the red straight line represents the kinematic-UCM and the green band (thin parallelogram) and blue dashed-line represent the D-UCM_anti_ and D-UCM_in_, respectively. The black curve exemplifies a sway trajectory for a single trial.

In each panel, the D-UCM_anti_ of the dynamic-UCM (depicted by the green parallelogram) appears as a nearly straight line. Additionally, the contribution rate of the principal component of the D-UCM_anti_, which corresponded to the direction of the line, was almost 1 (larger than 0.999) in both θ_a_-θ_h_ and ω_a_-ω_h_ planes for all subjects, meaning that the green parallelogram collapsed and was actually shaped as a straight line.

### Comparisons between the kinematic- and dynamic-UCMs

Remarkably, the D-UCM_anti_ of the dynamic-UCM (green parallelogram) was almost identical to the kinematic-UCM (red line) of every subject. Specifically, the kinematic-UCM was very similar to the D-UCM_anti_ in the θ_a_-θ_h_ plane for all subjects (Figures 4A1–A5). As quantified in Table 4, the kinematic-UCM slope and direction of the first principal component of the D-UCM_anti_ in the θ_a_-θ_h_ plane was about −3 for all subjects. Likewise, the kinematic-UCM was also very similar to the D-UCM_anti_ in the ω_a_-ω_h_ plane for all subjects, as shown in Figures 4B1–B5 (with a kinematic-UCM slope and D-UCM_anti_ principal component direction around −3). Together, these results revealed a nearly identical geometric arrangement between the kinematic-UCM and D-UCM_*anti*._ Further, our findings demonstrated that, at least with the resolution provided by the experimental measurement of postural sway, these two UCMs could not be distinguished from each other in either the θ_a_-θ_h_ or the ω_a_-ω_h_ plane.

**Table 4:** **Slope of the kinematic-UCM, directions of the first principal component for the dynamic-UCM, and postural sway trajectories in the θ_**a**_-θ_**h**_ and ω_**a**_-ω_**h**_ planes for each subject**.

	**Subject 1**	**Subject 2**	**Subject 3**	**Subject 4**	**Subject 5**
**SLOPE OF THE KINEMATIC-UCM IN THE θ_a_-θ_h_ AND ω_a_-ω_h_ PLANES**					
	−3.0820	−3.4645	−3.0395	−3.2534	−3.4039
**DIRECTION OF THE FIRST PRINCIPAL COMPONENT (*γ*_θ_ AND γ_ω_) AND THE CONTRIBUTION RATE**					
**Dynamic UCM (D-UCM_anti_)**					
γ_θ_	−2.60	−2.90	−2.53	−2.70	−2.86
(Contribution)	(>0.999)	(>0.999)	(>0.999)	(>0.999)	(>0.999)
γ_ω_	−2.53	−2.86	−2.49	−2.68	−2.81
(Contribution)	(>0.999)	(>0.999)	(>0.999)	(>0.999)	(>0.999)
**POSTURAL SWAY TRAJECTORY**					
**Original Data**					
γ_θ_	−4.91±8.82	1.47±1.47	0.50±5.77	5.77±14.78	0.20±0.8
(Contribution)	(0.81±0.097)	(0.77±0.111)	(0.79±0.066)	(0.72±0.119)	(0.76±0.067)
γ_ω_	−3.30±0.75	−3.24±0.27	−8.07±3.72	−3.15±0.41	−3.56±0.45
(Contribution)	(0.79±0.009)	(0.87±0.013)	(0.86±0.042)	(0.83±0.025)	(0.89±0.026)
**LOW-PASS FILTERED DATA (1-Hz CUT OFF FREQUENCY)**					
γ_θ_	−5.07±9.18	1.45±1.46	0.40±5.67	5.36±13.82	0.20±0.87
(Contribution)	(0.82±0.095)	(0.77±0.109)	(0.79±0.067)	(0.72±0.122)	(0.76±0.067)
γ_ω_	0.32±5.89	−1.95±0.54	5.46±14.77	−3.49±3.97	−2.38±0.72
(Contribution)	(0.60±0.071)	(0.69±0.052)	(0.77±0.100)	(0.62±0.053)	(0.75±0.044)
**HIGH-PASS FILTERED DATA (1-Hz CUT OFF FREQUENCY)**					
γ_θ_	−3.10±0.05	−3.44±0.11	−3.67±0.25	−3.12±0.14	−3.72±0.10
(Contribution)	(0.99±0.002)	(0.98±0.003)	(0.99±0.002)	(0.97±0.002)	(0.99±0.002)
γ_ω_	−3.53±0.07	−3.99±0.10	−3.72±0.13	−3.52±0.06	−4.54±0.12
(Contribution)	(0.99±0.000)	(0.99±0.001)	(0.99±0.001)	(0.99±0.001)	(0.99±0.001)

### Comparisons between kinematic- and dynamic-UCMs and postural sway trajectories

For the θ_a_-θ_h_ plane (Figures 4A1–A5), no clear relationship could be found between the original, non-filtered sway trajectory and the kinematic/dynamic-UCMs. This was also the case for the PCA (summarized in Table 4) that compared the directions of the kinematic/dynamic-UCMs with the first principal components for the original, non-filtered sway data in the θ_a_-θ_h_ plane, where the standard deviations of the eigenvector directions were large for most of the subjects. These results indicated that the joint angle trajectories in the θ_a_-θ_h_ plane did not exhibit any major orientation.

On the one hand, it is clear that angular velocity trajectories in the ω_a_-ω_h_ plane (Figures 4B1–B5) were located along the kinematic-UCM and D-UCM_anti_; however, the sway trajectory was not necessarily constrained by those manifolds, but rather spread widely around them. The PCA for the sway trajectory in the ω_a_-ω_h_ plane (Table 4) revealed that the direction of the first principal component was about −3, with small standard deviations for all subjects except Subject 3. Further, the contribution rate of the first principal component was very high, suggesting that the trajectory in the ω_a_-ω_h_ plane exhibited a specific orientation.

**Figure 5:**
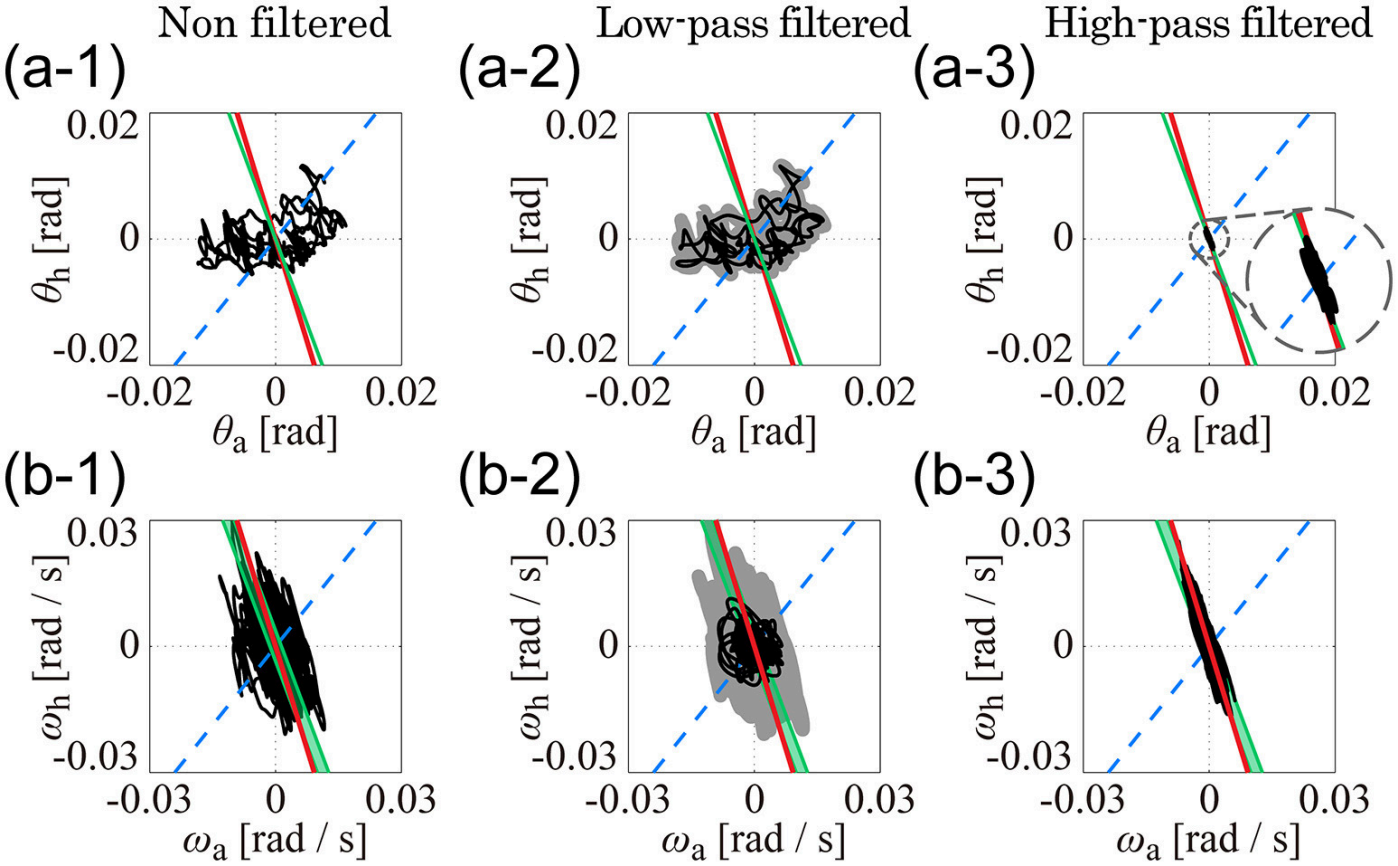
**Low- and high-pass filtered sway trajectories of ankle and hip joint dynamics during quiet stance (Subject 4). (A1–A3)** are for the θ_a_-θ_h_ plane and **(B1–B3)** are for the ω_a_-ω_h_ plane. See legend of Figure 4 for details of lines in each panel. **(A1,B1)** exhibit the non-filtered, original sway trajectories, which are exactly the same as **(A4,B4)** in Figure 4. Black curves in **(A2,B2)** are low-pass filtered (1-Hz cut off frequency) trajectories for the data in **(A1,B1)**. Gray thick curves represent the non-filtered, original trajectories, which are the same as the trajectories in **(A1,B1)**. Curves in **(A3,B3)** are the high-pass filtered trajectories for the data in **(A1,B1)** (1-Hz cut off frequency).

Figure 5 separates the roles of the low (Figures 5A2,B2) and high (Figures 5A3,B3) frequency sway trajectory components in the θ_a_-θ_h_ and ω_a_-ω_h_ planes, specifically for subject 1 (note that Figures 5A1,B1 are exactly the same as Figures 4A4, B4) but similar patterns characterize also the other subjects. Low-frequency components of the trajectory in the θ_a_-θ_h_ plane (Figure 5A2) were very similar to the original, non-filtered trajectory (Figure 5A1), implying that variations in the joint angles were dominated by low-frequency components below 1 Hz. On the other hand, low-frequency trajectory components in the ω_a_-ω_h_ plane (Figure 5B2) were distributed in a roughly circular shape and were much smaller in amplitude than the original, non-filtered trajectory (Figure 5B1). In both Figures 5A2,B2, no clear relationship could be found between low-frequency components of the trajectories and the kinematic/dynamic-UCMs. Contrastingly, in Figures 5A3,B3, high-frequency trajectory components were strictly located on the kinematic-UCM and D-UCM_anti_ in both the θ_a_-θ_h_ and ω_a_-ω_h_ planes. A comparison between high-frequency components in the θ_a_-θ_h_ plane (Figure 5A3) and the original trajectory (Figure 5A1) revealed that the amplitudes of the former were much smaller than the latter, but were characterized by a consistent linear relationship. Although the small amplitudes of the high frequency components suggest that they might be “negligible,” the strict relationship between these components and the kinematic-UCM and D-UCM_anti_ in both the θ_a_-θ_h_ and ω_a_-ω_h_ planes supports instead the hypothesis that components above 1 Hz play an important role in postural control during quiet stance.

Principal component analysis of the low-pass filtered trajectories in the θ_a_-θ_h_ and ω_a_-ω_h_ planes showed that the standard deviation of the eigenvector-direction was large for every subject, and that the mean slope values substantially varied, depending on the subject (Table 4: low-pass filtered data). These results quantitatively indicated that low-frequency components of the trajectories in the θ_a_-θ_h_ and ω_a_-ω_h_ planes were not aligned in a specific direction. On the other hand, high-pass filtered trajectories in both θ_a_-θ_h_ and ω_a_-ω_h_ planes exhibited specific orientations in the first principle component, with high contribution rates and very small standard deviations for all subjects (Table 4: high-pass filtered data). First eigenvector directions in the θ_a_-θ_h_ and ω_a_-ω_h_ planes were similar to each other, where mean ± SD values across subjects were −3.41 ± 0.29 for the θ_a_-θ_h_ plane and −3.86 ± 0.43 for the ω_a_-ω_h_ plane. These eigenvector directions were quite similar to the slope of the kinematic-UCM, as well as the direction of the first principal component of the D-UCM_anti_.

In summary, we found that there was no clear relationship between the non-filtered, original (also the low-pass filtered) trajectories and the kinematic/dynamic-UCMs in the θ_a_-θ_h_ plane, but that high-frequency components above 1 Hz (i.e., high-passed filtered trajectories) were aligned along the kinematic-UCM as well as the D-UCM_anti_. Moreover, angular velocity trajectories in the ω_a_-ω_h_ plane were also aligned along the kinematic-UCM and D-UCM_anti_.

### PSD analysis

**Figure 6:**
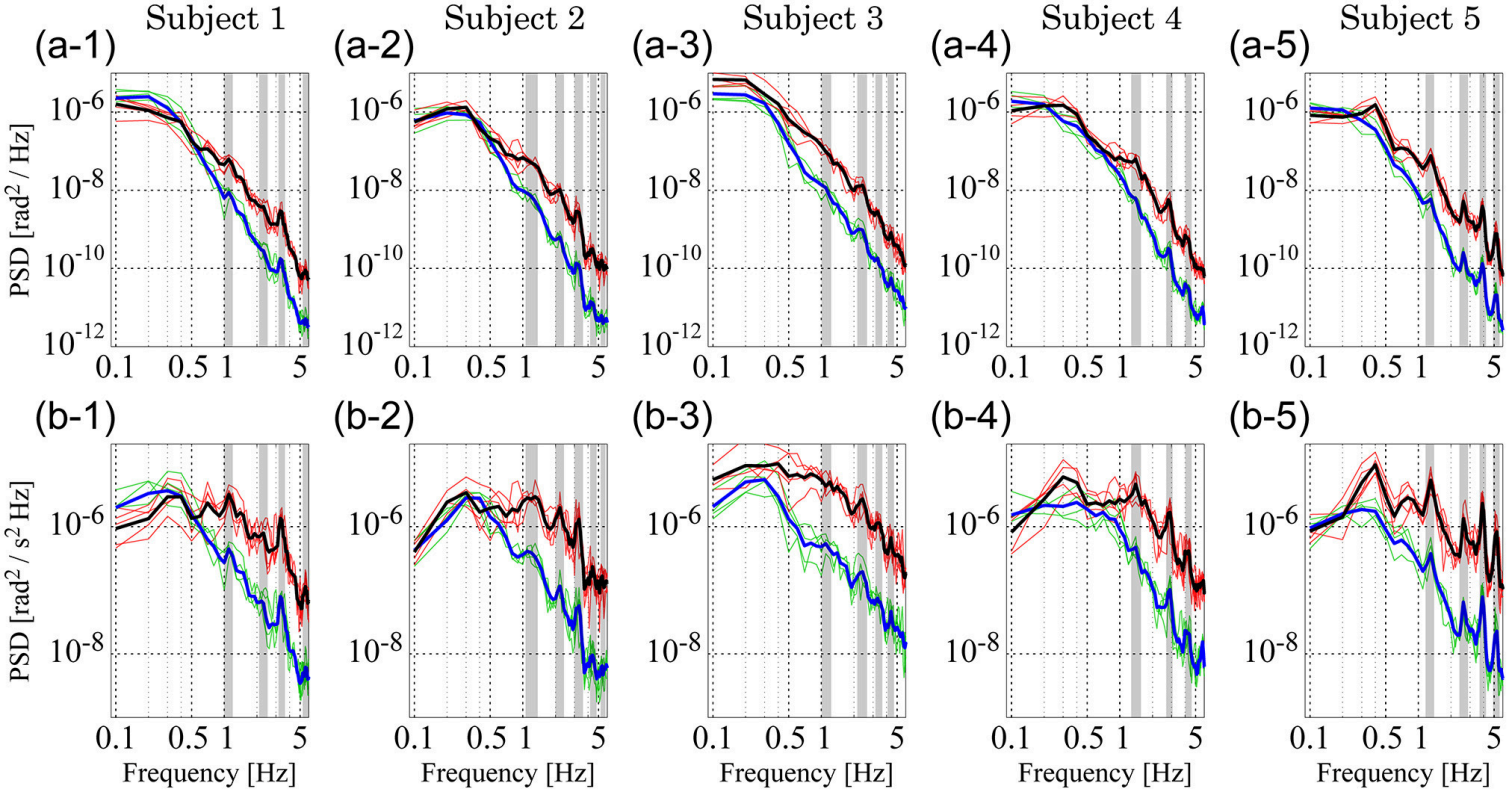
**Power spectrum density (PSD) functions of joint angular displacement and angular velocity for five subjects. (A1–A5)** Depict PSDs of joint angular displacement, while **(B1–B5)** exhibit those of angular velocity. In each panel, green and red curves represent PSDs of the ankle and hip joints for individual trials, respectively. Blue and black curves are the ensemble averages over trials of the green and red curves, respectively. Gray-shaded bars indicate the locations of peak frequencies between 1 and 6 Hz to characterize PSDs in the lower panels, which are extended into the upper panels to show the coincidence of peaks.

Figure 6 shows PSDs for the experimental sway data; PSDs of θ_a_ and θ_h_ (Figures 6A1–A5) and those of ω_a_ and ω_h_ (Figures 6B1–B5). In each panel, the thin green curves represent PSDs for ankle motion (θ_a_ in Figures 6A1–A5 and ω_a_ in Figures 6B1–B5) in the five trials, while the thick blue curve represents the ensemble average of those five ankle PSDs. The thin red curves represent the five sampled PSDs for hip motion (θ_h_ in Figures 6A1–A5 and ω_h_ in Figures 6B1–B5) and the thick black curve represents the ensemble average of those five hip PSDs.

Both ankle (blue) and hip (black) velocity PSDs exhibited several characteristic peaks in the high frequency regime above 1 Hz (Figures 6B1–B5). Peaks in each hip angular velocity PSD were determined and are indicated in the figure by gray-shaded bands. We found that the blue PSD (i.e., ankle angular velocity) exhibited characteristic peaks at the gray-shaded frequencies determined for the hip angular velocity. That is, PSDs for hip and ankle velocities exhibited characteristic peaks at the same frequencies. We extended the gray bands determined for Figures 6B1–B5 into Figures 6A1–A5 for the joint angles, and found that the PSDs for the ankle and hip joint angles also tended to exhibit characteristic peaks at the gray-shaded frequencies determined for the angular velocities. Table 5 summarizes those gray-shaded frequencies, where most of the characteristic peaks in the PSDs were integral multiples of the lowest characteristic frequency located at 1–1.5 Hz. Together, these findings reveal that peaks at higher frequencies were the harmonics of the fundamental frequency located at 1–1.5 Hz, suggesting that the ankle and hip joints oscillate at a characteristic frequency of 1–1.5 Hz.

**Table 5:** **Peak frequencies charactering power spectrum density (PSD); see Figure 6**.

**Subject no**.	**Fundamental frequency (: FF)**	**Frequency band**				
		**1–2 [Hz]**	**2–3 [Hz]**	**3–4 [Hz]**	**4–5 [Hz]**	**5–6 [Hz]**
1	1.1	1.1 (FF × 1)	2.3 (FF × 2)	3.3 (FF × 3)	—	5.6 (FF × 5)
2	1.1	1.1 (FF × 1)	2.2 (FF × 2)	3.2 (FF × 3)	4.4 (FF × 4)	5.4 (FF × 5)
3	1.1	1.1 (FF × 1)	2.4 (FF × 2)	3.3 (FF × 3)	4.4 (FF × 4)	—
4	1.4	1.4 (FF × 1)	2.8 (FF × 2)	—	4.2 (FF × 3)	—
5	1.3	1.3 (FF × 1)	2.6 (FF × 2)	3.9 (FF × 3)	—	5.3 (FF × 4)

### Eigenfrequency analysis

**Figure 7:**
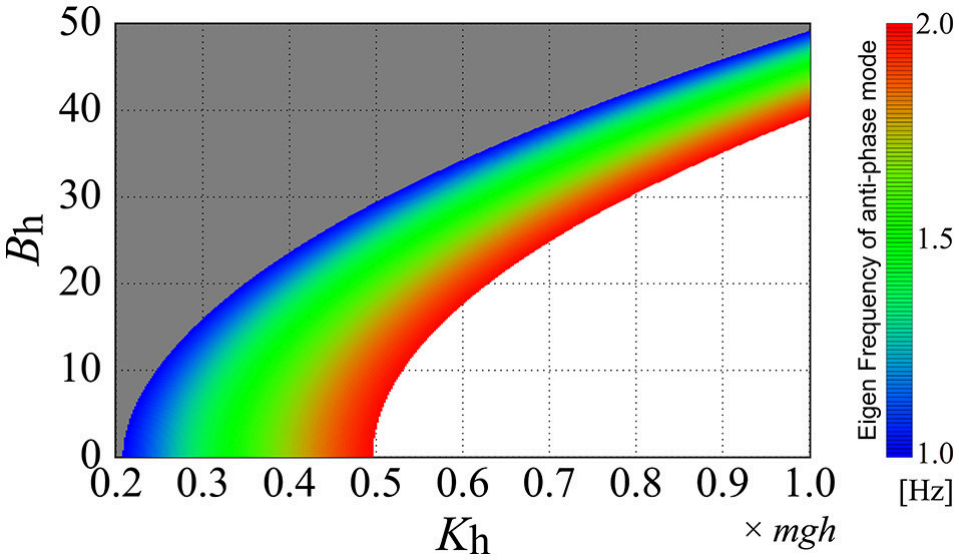
**Eigenfrequency of the anti-phase mode of the double inverted pendulum model without active feedback control (off-model) as a function of the passive elasticity ***K***_**h**_ and viscosity ***B***_**h**_**. The eigenfrequency at each set of (*K*_h_, *B*_h_) is color-coded based on colored bars on the right-side of the panel. The *K*_h_-*B*_h_ parameter regions are colored gray and white for eigenfrequencies lower than 1.0 Hz and higher than 2.0 Hz, respectively. In this figure (and throughout this study), the passive viscoelasticity at the ankle joint is fixed at (*K*_a_, *B*_a_) = (0.8*mgh*, 4.0).

Figure 7 shows how the eigenfrequency of the antiphase mode of the off-model, corresponding to the D-UCM_anti_, varies as a function of the passive elasticity *K*_h_ and viscosity *B*_h_ at the hip joint, where the eigenfrequency of the anti-phase mode is color-coded on the *K*_h_-*B*_h_ plane. Based on our finding that the fundamental frequency of anti-phase joint motion during quiet standing was 1–1.5 Hz (Table 5), we focused on the eigenfrequency of the anti-phase mode between 1 and 1.5 Hz by coloring the parameter regions for the eigenfrequency below 1.0 Hz in gray and that above 2.0 Hz in white. We found that the larger the passive hip elasticity, *K*_h_, the higher the eigenfrequency of the anti-phase mode of the off-model. On the other hand, the larger the passive hip viscosity, *B*_h_, the lower the eigenfrequency. The parameter region corresponding to the eigenfrequency of the anti-phase mode of the off-model (i.e., close to the experimental fundamental frequency) is represented by the color-band between blue to blue-green, showing the relatively wide distribution range of this eigenfrequency in the parameter space. In other words, if the passive viscoelasticity of the hip joint were to be located within this parameter region, the off-model—in the absence of active feedback control—would be able to exhibit anti-phase, ankle-hip coordination with frequencies consistent to those of our experimental observation.

### Eigenfrequency and stability of the anti-phase mode of the on-model

Although the off-model without active feedback control can exhibit anti-phase, ankle-hip coordination with frequencies consistent to those of our experimental observation, there remains a possibility that the on-model with time-delayed, active feedback control can also exhibit anti-phase, ankle-hip coordination of similar frequencies. If this is the case, then anti-phase coordination near the D-UCM_anti_ may not necessarily be associated with the off-model. In the paragraph that follows, we discuss how the on-model can just barely exhibit anti-phase, ankle-hip coordination with frequencies from our experimental observation. Further, such coordination in the on-model can only occur when the combination of passive and active feedback gain values are tuned to within a very narrow parameter region.

**Figure 8:**
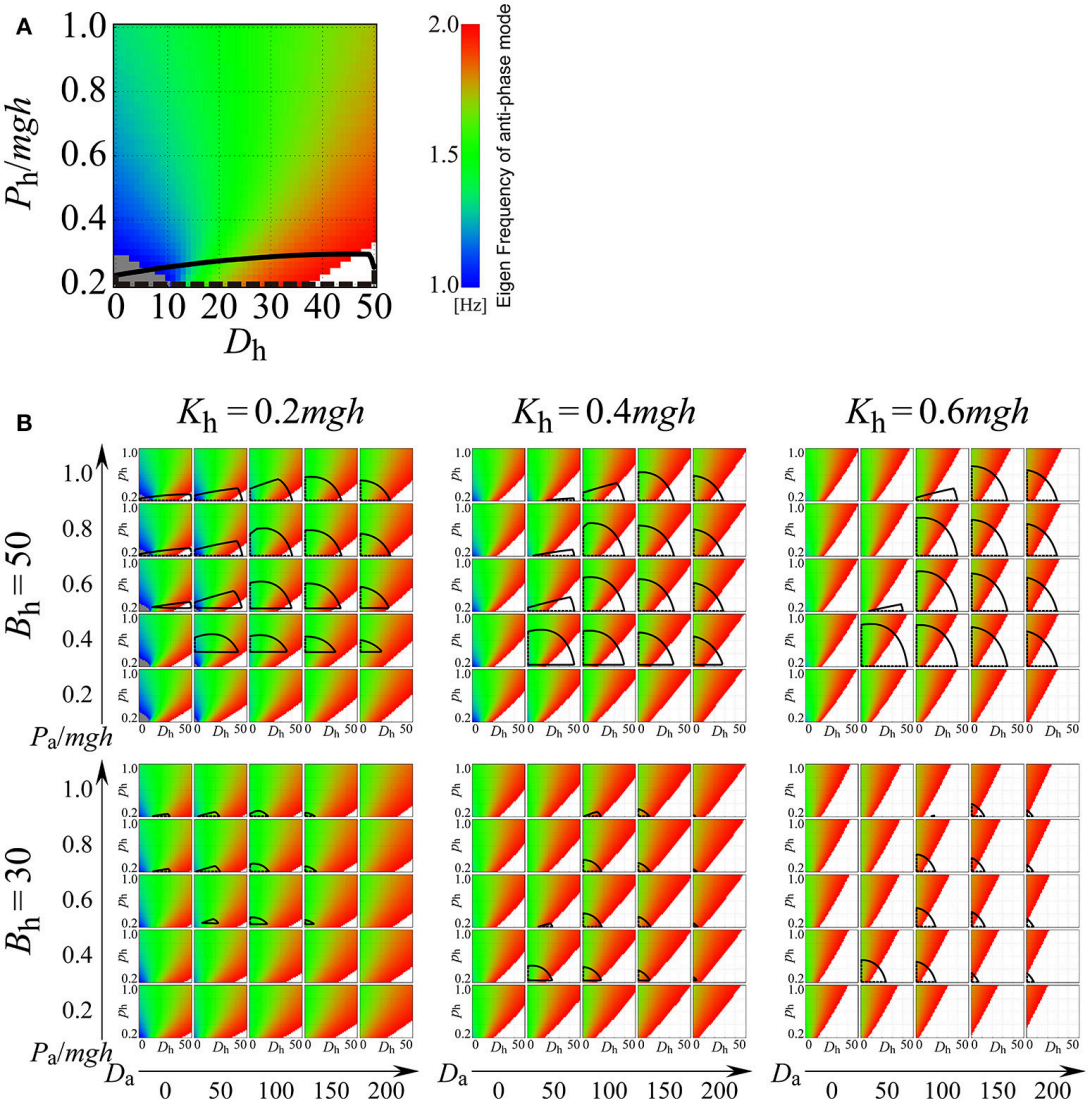
**Eigenfrequency and stability of the anti-phase mode for the double inverted pendulum model with continuous active feedback control (on-model) on the ***D***_**h**_-***P***_**h**_ parameter plane for varied parameter values of ***K***_**h**_, ***B***_**h**_, ***D***_**a**_, and ***P***_**a**_, where ***D***_h_ and ***P***_**h**_ are the derivative and proportional gains of the active feedback controller at the hip joint, respectively, and ***D***_**a**_ and ***P***_**a**_ are the derivative and proportional gains of the active feedback controller at the ankle joint, respectively**. See legend of Figure 7 for color identification details. In each panel, the area surrounded by the black curves represents the stability region. **(A)** Example case (*K*_h_, *B*_h_, *P*_a_, *D*_a_) = (0.2*mgh*, 50, 1.0*mgh*, 0). **(B)** Systematic exploration for various values of *K*_h_, *B*_h_, *D*_a_, and *P*_a_. In this study, the passive viscoelasticity at the ankle joint was fixed at (*K*_a_, *B*_a_) = (0.8*mgh*, 4.0).

Figure 8 explores how the eigenfrequency of the anti-phase mode in the on-model changes depending on the values of passive viscoelasticity (*K*_h_ and *B*_h_) and proportional and derivative gains at the ankle joint (*P*_a_ and *D*_a_), as well as proportional and derivative gains of the active, delayed feedback controller (*P*_h_ and *D*_h_) at the hip joint. In Figure 8, in addition to the eigenfrequency of the anti-phase mode, stability regions of the on-model are also clarified (the region surrounded by the black line in each *P*_h_-*D*_h_ plane). For example, Figure 8A depicts the exploration of the *P*_h_-*D*_h_ parameter plane when the passive elastic and viscosity coefficients of the hip joint are fixed at (*K*_h_, *B*_h_) = (0.2*mgh*, 50) and the proportional and derivative gains of the active feedback controller at the ankle joint are fixed at (*P*_a_, *D*_a_) = (1.0*mgh*, 0), where, as in Figure 7, the *P*_h_-*D*_h_ parameter plane is colored by the eigenfrequency of the anti-phase mode. The region with the color band between blue and red represents the eigenfrequency between 1.0 and 2.0 Hz, while the gray and white regions indicate the eigenfrequency below 1.0 Hz and above 2.0 Hz, respectively. However, Figure 8A is a singular case, where passive elasticity at the hip is very small (*K*_h_ = 0.2*mgh*) and passive viscosity at the hip is relatively large (*B*_h_ = 50 Nms/rad). In this case, the on-model can exhibit the anti-phase mode with a frequency between 1.0 and 1.5 Hz for *D*_h_, below about 15 Nms/rad (the region colored by blue or blue-green), which spans a relatively wide region. However, even in the above-described case, we postulate that the D-UCM_anti_ of the off-model is responsible for the experimentally observed anti-phase coordination for the following reasons. In our framework (Figure 8A), we contrasted two control mechanisms that could stabilize upright posture with anti-phase, ankle-hip coordination with a frequency between 1.0 and 1.5 Hz. The first mechanism (continuous control) was characterized by the on-model being persistently utilized, whereby upright posture was solely stabilized by the active feedback controller (i.e., the on-model without switching between on- and off-models). In this case, *P*_h_-*D*_h_ parameter values should be located within the stability region, and the *P*_h_-*D*_h_ region should be blue-green in color such that it exhibits anti-phase, ankle-hip coordination with a frequency between 1.0 and 1.5 Hz. As can be seen, overlap between these two regions was very small (*P*_h_ was close to 0.2*mgh* and *D*_h_ between 10 and 15 Nms/rad); thus, it is physiologically implausible and less likely that *P*_h_-*D*_h_ parameter values are finely tuned to such small region. The other mechanism that we utilized in our framework was the intermittent feedback control strategy, where the feedback controller switched between the off- and on-model. Note that each off- and on-model is typically unstable in the intermittent control model (Suzuki et al., 2012). In our sample case, use of the off-model was inevitable for the stability of upright posture (i.e., upright posture could not be stabilized without the off-model), implying that the D-UCM_anti_ of the off-model was responsible for the experimentally observed anti-phase coordination.

Figure 8B includes six panels with different combinations of passive viscoelasticity (*K*_h_, *B*_h_; fixed in each panel); each panel consists of 25 *P*_h_-*D*_h_ planes for varying arrangements of active feedback gains at the ankle (*P*_a_, *D*_a_; fixed in each plane). For most combinations of active feedback gain at the hip (*P*_h_, *D*_h_) and ankle (*P*_a_, *D*_a_), the blue or blue-green regions appear quite limited (Figure 8B), revealing that the on-model hardly exhibits the experimentally observed anti-phase, ankle-hip coordination of 1.0–1.5-Hz frequencies. Keeping this observation in mind, and exploring Figure 8B in more detail, it can be seen that the 25 *P*_h_-*D*_h_ planes in the panels for *K*_h_ = 0.6*mgh* (corresponding to large, passive stiffness at the hip), with a *B*_h_ of either 30 or 50 Nms/rad and in the panel for (*K*_h_, *B*_h_) = (0.4*mgh*, 30), exhibit wide red (and white) regions. This observation suggests that the eigenfrequency of the anti-phase mode was higher than 1.5 Hz, and that it could therefore not be tuned to frequencies between 1 and 1.5 Hz. Thus, we concluded that the on-model would never be responsible for the experimentally observed anti-phase, ankle-hip coordination occurring between frequencies of 1.0 and 1.5 Hz, regardless of active feedback gain values at the hip (*P*_h_, *D*_h_) for sets of (*K*_h_, *B*_h_). In other words, if the physiological values of (*K*_h_, *B*_h_) fall within the above-indicated ranges, anti-phase, ankle-hip coordination of frequencies between 1.0 and 1.5 Hz should be generated by the off-model.

The panels for (*K*_h_, *B*_h_) = (0.2*mgh*, 30), (*K*_h_, *B*_h_) = (0.2*mgh*, 50), and (*K*_h_, *B*_h_) = (0.4*mgh*, 50) comprise of some *P*_h_-*D*_h_ planes that involve blue or blue-green regions, meaning that, as in Figure 8A, the on-model can exhibit anti-phase, ankle-hip coordination with a frequency between 1.0 and 1.5 Hz, but only if *P*_h_ and *D*_h_ are set to small values. However, stability regions of the on-model, which span relatively wide areas around the middle of the *P*_h_-*D*_h_ planes in the three panels, do not overlap with either the blue or the blue-green regions. Thus, the eigenfrequency of the anti-phase mode in the stable on-model is much higher than the 1−1.5 Hz band. Since small *P*_h_ and *D*_h_ values in the unstable on-model require switching to the off-model for upright posture stabilization, once again, we concluded that the D-UCM_anti_ of the off-model was responsible for the experimentally observed anti-phase coordination.

Together, these results suggest that the experimentally observed anti-phase coordination between the hip and ankle joints was generated by the dynamics along the off-model D-UCM_anti_ (without active feedback control), and that this was the case regardless of the passive viscoelasticity values (*K*_h_ and *B*_h_).

## Discussion

### Summary

In this study, we considered two types of uncontrolled manifolds (kinematic- and dynamic-UCMs) during human quiet stance based on a double inverted pendulum model of the human body. The kinematic-UCM is defined by a kinematic constraint such that the CoM of the whole body is constant in the anterior-posterior direction (Creath et al., 2005; Hsu et al., 2007; Pinter et al., 2008). On the other hand, the dynamic-UCM is defined by the stable manifold of a saddle-type unstable upright equilibrium of the double inverted pendulum with no active feedback control (the off-model), in association with an intermittent feedback control hypothesis postulating that the CNS stabilizes upright posture by intermittent and appropriate inactivation of a feedback controller (Bottaro et al., 2008; Asai et al., 2009; Suzuki et al., 2012). Here, we showed that the kinematic- and dynamic-UCMs (particularly, the two-dimensional stable manifold denoted by D-UCM_anti_ associated with the oscillatory stable mode of the unstable dynamics exhibiting anti-phase coordination between the ankle and hip joints) were almost identical, meaning that the geometrical configuration of these two types of UCM in the ankle-hip joint angle space (θ_a_-θ_h_ plane) and in the ankle-hip angular velocity space (ω_a_-ω_h_ plane) were quite similar to each other.

We plotted experimentally observed postural sway on the θ_a_-θ_h_ and ω_a_-ω_h_ planes, and showed that the high-frequency component (>1 Hz) varied along either kinematic- or dynamic-UCMs (because of their similarity), but that the low-frequency component (<1 Hz) spanned the subspace complemental to the UCM. A spectral analysis using PSDs of the postural sway data showed that the PSD of every subject exhibited a characteristic peak, which represented the fundamental frequency in the 1.0−1.5-Hz band and its harmonics. Moreover, this fundamental frequency coincided with the eigenfrequency of the anti-phase mode, which corresponded to the dynamics on the D-UCM_anti_ of the off-model. In order to confirm that the oscillation with the anti-phase coordination at 1.0−1.5 Hz was generated by the anti-phase mode of the off-model, we examined whether the on-model could also generate an anti-phase oscillation (either stable or unstable) with a fundamental frequency of 1.0−1.5 Hz. Our analysis of the on-model employed a wide range of passive viscoelasticity values and active delayed feedback controller gains, and revealed that the parameter regions for the on-model that could exhibit the experimentally observed anti-phase coordination (within a range of 1.0−1.5 Hz) were extremely limited (Figure 8). Thus, the experimentally observed, anti-phase coordination was most likely generated by dynamics of the off-model along the dynamic-UCM. Moreover, these findings support the intermittent feedback control hypothesis, which postulates that coordinated motion between the ankle and hip joints is caused by purely mechanical, passive dynamics of the human body with smart and intermittent use of off-model dynamics by the CNS to stabilize upright posture during quiet stance.

### Passive dynamics of the human body are responsible for ankle-hip coordination

The results summarized above are consistent with a study by Saffer et al. (2008) that reported a correlation between ankle muscle activation patterns and leg posture during quiet stance (reflecting counteractivity to gravity), but no clear correlation between trunk segment posture and relevant muscle activation. Based on these results, they concluded that anti-phase coordination between the trunk and lower extremities at high frequency bands had arisen from indirect (i.e., passive), biomechanical control of the posterior leg muscles.

In our previous study, we reported that angular accelerations of the ankle and hip joints (denoted by α_a_ and α_h_) exhibit a strong correlation with a negative correlation coefficient (referred to as the reciprocal relationship), and that trajectories representing postural sway dynamics in the α_a_-α_h_ plane are located along a specific subspace of the α_a_-α_h_ plane (referred to here as the UCM_a_) such that acceleration in the anterior-posterior direction of CoM is zero. Although such a simple reciprocal relationship seemingly reflects underlying active control, we revealed that the reciprocal relationship is always held by Newton's second law for the double inverted pendulum, and that this is true regardless of the passive (as well as the active) joint torque patterns acting on the ankle and hip joints (Suzuki et al., 2015). In this study, we showed that joint angles and angular velocities at the ankle and hip joints exhibited anti-phase, coordinated oscillations at a fundamental frequency of 1−1.5 Hz. This anti-phase coordination corresponded to the characteristic frequency of the power spectra for angular accelerations at the ankle and hip joints shown in our previous study, implying that the anti-phase coordination examined in this study resembled the reciprocal relationship.

Zhang et al. (2007) analyzed coordinated motion between lower extremities and the upper body during quiet stance under several instances of varying visual (eyes-open/eyes-closed) and tactile (with/without light-touch) information. Using this approach, they observed a reduction in in-phase coordination between the ankle and hip joints (with frequencies below 1 Hz) for the eyes-open and light-touch conditions, while anti-phase coordination above 1 Hz did not change for any condition. Their results suggest that sensory information does not exert great influence over anti-phase coordination between the ankle and hip joints, and this finding is consistent with our current study, which showed that passive dynamics of the body without active control were responsible for anti-phase coordination.

Thus, passive dynamics of the human body without active feedback control seem to be responsible for ankle-hip coordination, which appear be negatively correlated with angular accelerations between the ankle and hip joints. Together with the intermittent feedback control scenario, postural sway during quiet stance can be formulated as follows: When the postural state point is located on/near the dynamic-UCM during which the active feedback control is inactivated (off-model), purely mechanical, passive dynamics are responsible for sway, and the state point oscillates on/along the dynamic-UCM (transient convergence toward the upright position). Since the dynamic-UCM is equivalent to the stable anti-phase mode of the off-model, resulting angular accelerations at the ankle and hip joints exhibit a reciprocal relationship, as shown in our previous study. Further, due to the unstable nature of the upright position of the off-model, as the state point transiently approaches the upright position, it eventually begins to move away from the dynamic-UCM, at which point the active feedback controller is switched on (on-model). As shown in Suzuki et al. (2012), the on-model in the intermittent feedback control model is also unstable due to delay-induced instability, where the unstable, in-phase mode (rather than the unstable, anti-phase mode) plays a significant role in kicking the falling pendulum upward. This in turn provides an opportunity for the state point to be close to the dynamic-UCM, which triggers a subsequent switch to the off-model. Indeed, the unstable, in-phase mode of the on-model exhibits slow dynamics, which correspond to the low frequency component (<1 Hz) in experimental postural sway; however, it should be noted that a quantitative analysis of the low frequency component of in-phase coordination is beyond the scope of the current study.

Taking the above conclusion into consideration, it is worth mentioning the different dynamics of postural sway between healthy young and elderly subjects, particularly in relation to ankle-hip coordination. Kato et al. (2014) reported that CoM variation in the elderly is significantly larger than in young subjects. Interestingly, they revealed that elderly individuals exhibit a reduction in anti-phase coordination amplitude when compared to that of young subjects. These results can be interpreted as follows. First, a reduction of anti-phase coordination in the elderly might be caused by an increase in passive viscoelasticity at the hip joint, since a larger passive viscoelasticity induces small-amplitude anti-phase oscillations at frequencies much higher than those observed experimentally (Figures 6–8). Moreover, an increase in the range of CoM variation in the elderly results in an increase in the in-phase (as opposed to anti-phase) coordination, since in-phase coordination between the trunk and lower extremities is always accompanied by a CoM-shift. The logical consequence is that aging may worsen the ability of elderly individuals to adjust their posture close to the kinematic- and dynamic-UCMs, namely a problem of deteriorated control efficiency. A number of different explanations of such deterioration may be formulated: (1) increased delay of the sensorimotor feedback loop; (2) increased noise of the sensory information used by the brain for detecting the location of one's own body dynamics in the phase space and/or the distance of the postural state from the kinematic- and dynamic-UCMs; (3) increased uncertainty of the switching mechanism implied by the intermittent controller. Such explanations are not necessarily alternative and they mix their effects in a variety of manners, specifically for each individual.

### The kinematic- vs. dynamic-UCM; cooperative roles played by two different control strategies

The geometrical similarity between the kinematic- and dynamic-UCMs implies that it is difficult to determine which strategy, namely the CoM-control hypothesis based on the kinematic-UCM or the intermittent feedback control hypothesis based on the dynamic-UCM, is more physiologically plausible in its use by the CNS for upright stance stabilization. Nevertheless, the current study support the hypothesis that the CNS stabilizes upright posture by using the intermittent feedback control strategy, in which the CNS exploits the transiently converging dynamics onto the dynamic-UCM and thus toward the unstable upright position.

The following discussion is centered on possible mechanisms of how the postural state point can move near the kinematic- and dynamic-UCMs, at least for some periods of time. First, it is important to point out that the CoM-control strategy is necessarily forced to utilize an active feedback controller that operates continuously, rather than intermittently, in order to reduce the sway component complemental to the kinematic-UCM. As a reminder, the kinematic-UCM is defined based on the gravitational balance among human body segments, and thus this UCM depends on body parameters and the geometrical relationship among CoM positions of the segments. Because of this, the kinematic-UCM cannot determine dynamics on the UCM nor the stability of upright posture, even if dynamics of the system are only considered for the state point on or close to the kinematic-UCM. Hence, without some mechanism to push the postural state point closer to the kinematic-UCM (i.e., a continuously active feedback controller), the postural state point would easily deviate from the kinematic-UCM. In fact, this deviation would even occur if the postural state point were located exactly on the kinematic-UCM.

For the CoM-control hypothesis, there are several possible strategies that can fulfill the requirement of a continuous, active feedback controller to facilitate the state point to move along the kinematic-UCM. One such strategy is the sliding mode control (SMC) (Utkin, 1977; Young et al., 1999; Zhang et al., 2016). A model with the SMC requires a low-dimensional hyperplane that includes a desired state point (upright posture in the case of postural control), referred to as the sliding surface. The sliding surface is designed so that the desired state point is asymptotically stable. If we consider the kinematic-UCM as the sliding surface, upright posture would not be a stable equilibrium point of the subsystem governing dynamics along the kinematic-UCM. This is because the kinematic-UCM is defined based on the geometry of the CoM, without taking the stability of the equilibrium point into account. In this way, the kinematic-UCM could not be the sliding surface (in terms of the SMC) without introducing a control mechanism for stabilizing upright posture. Contrastingly, the dynamic-UCM by itself could serve as a sliding surface because it is a stable manifold of a saddle-type, unstable equilibrium point; thus, the state point on the dynamic-UCM would approach the upright posture without any help of active control mechanisms.

One may argue that the intermittent feedback control hypothesis (Asai et al., 2009; Suzuki et al., 2012) is the CoM-control hypothesis with additional controllers for stabilizing upright posture, as it also utilizes an active feedback controller to ensure the state point to be close to the dynamic-UCM. However, the action of the active feedback controller used for the on-model in the intermittent control hypothesis is quite different from feedback controllers used in the typical SMC, although it aims at directing the UCM as the sliding surface. The difference is apparent in the on-off switching frequency of the active feedback controller. If the SMC is used for the CoM-control hypothesis, the feedback controller might be designed so that it forcefully directs the kinematic-UCM as the sliding surface, which typically causes high-frequency chattering. This is because the kinematic-UCM does not possess by itself any specific dynamic mechanism to keep the state point close to the kinematic-UCM, as discussed above, and thus the state point would rapidly deviate from the sliding surface without forceful feedback actions. Together with the fact that the kinematic-UCM cannot induce by itself any dynamics that can be associated with anti-phase coordination, this suggests that the CoM-control hypothesis implemented with the SMC is not capable of exhibiting the experimentally observed postural sway along the kinematic-UCM (Figure 6, Table 5).

Contrastingly, the active feedback controller in the intermittent control strategy typically utilizes delay-induced, unstable, oscillatory dynamics which does not aim at forcing the state point to approach the dynamic-UCM. Instead, such unstable, oscillatory dynamics around the upright equilibrium naturally provides an opportunity for the state point to approach the dynamic-UCM within a period of time after the active controller is switched on. Moreover, the dynamic-UCM itself does involve dynamics to move the state point along the dynamic-UCM, as discussed above; thus, once the active control is switched off, the state point of the off-model can move on the sliding surface without feedback. For these reasons, the on-off frequency in the intermittent control strategy is typically far less than that in the SMC, providing opportunities for the state point to exhibit anti-phase oscillations in the dynamic-UCM for certain periods of time.

Taken together, and in terms of the major mechanism of anti-phase coordination between the hip and ankle joints along the UCM during quiet standing, it is natural to conclude that the CNS utilizes transiently converging dynamics on the dynamic-UCM (via the intermittent feedback control strategy) rather than the kinematic-UCM (via the CoM-control strategy). Nevertheless, the results of this study also suggest that the CoM-control hypothesis and the intermittent feedback control hypothesis are closely related to each other, and that the strong similarities between the kinematic- and dynamic-UCMs provide an opportunity for the CNS to simultaneously establish a small range of CoM variations and robust bounded stability. In summary, the goal of the active controller in the CoM-control strategy is to drive the postural vector close to the kinematic-UCM, whereas that in the intermittent control strategy is to drive the postural state close to the dynamic-UCM. Both hypothetical strategies assume that the CNS suspends the active control when the postural vector or the state is near the UCM. Because of the geometric similarity between the kinematic- and dynamic-UCMs, inactivations of the active control turn out to be occurring at similar timings in both hypotheses. These aspects are the similarity between the CoM-control hypothesis and the intermittent control hypothesis. The difference between two hypotheses is that the CoM-hypothesis alone cannot explain how the upright posture (even if the postural vector is on the kinematic-UCM) is stabilized, but it can explain, by definition of the kinematic-UCM, how the CoM variations are reduced, whereas the intermittent control hypothesis can explain how stability of the upright posture is achieved using transiently converging dynamics on the dynamic-UCM. Although the dynamic-UCM is not defined in relation to constancy of the CoM, the dynamic characteristics of the dynamic-UCM, i.e., the oscillatory anti-phase mode associated with the dynamic-UCM, can make CoM variations small, implying that the CoM-control hypothesis can be considered as part of the more general intermittent control hypothesis.

### Relations to other studies

Gawthrop et al. proposed an intermittent control model, which includes a different type of intermittency (Gawthrop et al., 2011; Loram et al., 2011) from the intermittent feedback control hypothesis (Bottaro et al., 2008; Asai et al., 2009; Suzuki et al., 2012). They also investigated similarities and differences between the intermittent feedback control model referred to as Zero Control (ZC) and their intermittent control model (Gawthrop et al., 2014), referred to as the Open-Loop Trajectory (OLT). In the OLT model, a “predictor” is placed in the feedback loop for compensating the feedback time delay, where the predictor intermittently samples the postural state. According to the intermittently sampled state, an internal model performs estimations of the current and predicted states until the next sampling time, and these estimations are used for calculating the active control torque. Note that, in the OLT model, the postural state is observed intermittently, while the active control torque is provided continuously. On the other hand, in the ZC model, the active “feedback” control is provided intermittently, while the (delayed) postural state is observed continuously. Although simulated postural sway in a single inverted pendulum model of the OLT is similar to that of the ZC in state space, we expect that intermittent control with the OLT model (if applied to the double inverted pendulum model) might not be able to explain postural sway dynamics along the UCM with an anti-phase coordination between 1 and 1.5 Hz as continuous control is not appropriate for producing such dynamics.

Insperger et al. have considered the continuous proportional-derivative-acceleration (PDA) controller to include a predictor-like mechanism by introducing acceleration feedback (Insperger et al., 2013; Insperger and Milton, 2014), and have shown expansion of the stability region in comparison with the PD-feedback controller without acceleration-feedback controller. However, as in the case of the OLT, the continuous PDA control model (if applied to the double inverted pendulum model) might not be able to explain postural sway dynamics along the UCM with anti-phase coordination between 1 and 1.5 Hz because of a lack of intermittency in the feedback action. Moreover, in both cases the crucial role attributed to the prediction mechanism is by itself a cause of reduced robustness if we consider the physiological level of noise of proprioceptive signals. In contrast, the intrinsic robustness of the intermittent control strategy is based on the simplicity of the switching mechanism and its capability to tolerate the large delay of sensory feedback.

Generally speaking, achieving stability via prediction for a system that is strongly affected by noise and intrinsic instability is a biologically implausible strategy, or is at least less plausible than a strategy that exploits self-stabilization features of whole-body biomechanics.

### Limitations of the current study

#### Validation of the similarity between the kinematic- and dynamic-UCMs

One may argue whether the similarity between kinematic- and dynamic-UCMs can be validated or quantified statistically. Unfortunately, however, these two spaces are defined based on the mathematical models, not based on statistical properties that are derived from experimental sway data. Since they are mathematically different spaces, having even different dimensionalities, rigorously speaking, they cannot be even compared whether they are the same or not, and thus they can never be identical. In this sense, we have just stated that these two spaces are similar with each other qualitatively. This is also the case for comparison between theoretical values of UCM slope and the slopes of the principal axis that were obtained from the experimental sway data. The values of UCM slope calculated from the models are not distributed statistically, but they are given as single values deterministically by the equations of motion, although they are parameterized by individual body-mass parameters. If the double-inverted-pendulum model were not an approximation but were a true description of the body dynamics, the experimentally obtained principal axis may distribute around the theoretical value. However, it is not the case in this study unfortunately.

#### Generalizability of the results

In this study, a comparison was made between kinematic- and dynamic-UCMs based on the double-inverted-pendulum model, and we showed similarity between them. However, it is of critical importance to made a further comparison using multi-link-inverted-pendulum models that include more joints, such as knee and neck joints, with three or more links, and examine whether similarity could also be observed in multi-link-inverted-pendulum models, because it is reported that dominant postural sway can be better explained by the kinematic-UCM of a six-link-inverted-pendulum than that of a double-inverted-pendulum (Hsu et al., 2007). Although such comparison is outside the scope of this study, the analytical technique that maps two or three dimensional plane representing the stable manifold of the upright saddle-point in the four-dimensional dynamic state space for the double-inverted-pendulum model into the two-dimensional kinematic joint angle space can always be applicable to multi-link-pendulum models, and it should be considered as a future issue. In case of six-link-inverted-pendulum model in the sagittal plane, the dimension of the state space becomes twelve, for which a certain amount of calculations to find eigenvalues of a twelve-dimensional differential equation is required to obtain the dimension of its stable mode. Interestingly, Tanabe et al. (2016) showed that the intermittent feedback control can stabilize a quadruple inverted pendulum model, which implies a stable manifold of the quadruple inverted pendulum model without active control occupies a high-dimensional subspace of the eight-dimensional state space of the model. If the results of this study can be generalized for multi-link pendulum models, such a high-dimensional stable manifold, or its subspace dominating the stability, it is expected that the stable manifold will be similar to the high-dimensional kinematic-UCM.

Gender and age dependency of postural swayIt could be problematic that the subjects participated in this study were all young male adults, since postural sway and thus postural control strategy can be age-dependent (see Demura et al., 2008; Oba et al., 2015, for example). However, there is also a report showing that no gender effect and interaction exist in anterior-posterior sway (Kim et al., 2010). In any case, it should be discussed as a future issue of how gender and age could alter the results shown in this study.

### Remarks

In this paper and in our previous related studies, we assume the “off” parameters (“passive” joint stiffness and viscosity during off-phases) are constant over time. However, in fact, they are somewhat controlled by the CNS, i.e., co-activation levels of agonistic-antagonistic and stabilization muscles will alter the apparent “passive” stiffness and viscosity of the joints. Therefore, in reality, even in the off-phases of the control, “passive” joint viscoelasticity (i.e., joint viscoelasticity that do not alter directly depending on the on-off switching of the active feedback controller) is actually modulated by the CNS, but at a different time scale (slower time scale). Such a slow modification may also be affected by a feedforward controller, and altered by environmental conditions as well as motor learning.

## Author contributions

Designed research: YS, PM, TN. Experimental data acquisition: YS, HM. Mathematical model formulation, analysis and simulation: YS. Time-series data analysis: YS, HM, KK. Wrote paper: YS, PM, and TN.

## Funding

This work was supported in part by JSPS grants-in-aid 26242041 and 26750147, MEXT KAKENHI Grant Number 16H01614 (Non-linear Neuro-oscillology), and MEXT grant for “post-K project (primary issue 2)”.

### Conflict of interest statement

The authors declare that the research was conducted in the absence of any commercial or financial relationships that could be construed as a potential conflict of interest.
