# Supplementary material for: Dynamic Determinants of the Uncontrolled Manifold during Human Quiet Stance
Source: Front Hum Neurosci. 2016 Dec 6;10:618. doi: 10.3389/fnhum.2016.00618 (PMC5138220; doi:10.3389/fnhum.2016.00618)
Supplement: Supplementary file 1 [file DataSheet1.pdf]

Appendix A: Model definition

The matrices and vectors in Eq. (2) are defined as follows:

$$\boldsymbol{\theta} = \begin{pmatrix} \theta_a \\ \theta_h \end{pmatrix}, \tag{A.1}$$

$$\mathbf{M} = \begin{pmatrix} I_L + I_{HAT} + m_{HAT} l_L^2 + 2 m_{HAT} l_L h_{HAT} & I_{HAT} + m_{HAT} l_L h_{HAT} \\ I_{HAT} + m_{HAT} l_L h_{HAT} & I_{HAT} \end{pmatrix}, \tag{A.2}$$

$$\mathbf{G} = \begin{pmatrix} -g m_L h_L - g m_{HAT} l_L - g m_{HAT} h_{HAT} & -g m_{HAT} h_{HAT} \\ -g m_{HAT} h_{HAT} & -g m_{HAT} h_{HAT} \end{pmatrix}, \tag{A.3}$$

$$\mathbf{Q} = \begin{pmatrix} \tau_a \\ \tau_h \end{pmatrix}, \tag{A.4}$$

where g represents gravitational acceleration. I_L and I_{HAT} represent the inertia moment of the lower link around the ankle joint and that of the upper link around the hip joint, respectively. τ_a and τ_h represent joint torques at ankle and hip joints. Joint torque $\mathbf{Q} = (\tau_a, \tau_h)^T$ is described as the linear sum of the passive joint torque $\mathbf{Q}_p = (\tau_a^{passive}, \tau_h^{passive})^T$ without time delay and active joint torque $\mathbf{Q}_a = (\tau_a^{active}, \tau_h^{active})^T$, which involves time delay (Asai et al. 2009; Suzuki et al. 2012).

$$\begin{aligned} \begin{pmatrix} \tau_a \\ \tau_h \end{pmatrix} &= \begin{pmatrix} \tau_a^{passive} \\ \tau_h^{passive} \end{pmatrix} + \begin{pmatrix} \tau_a^{active} \\ \tau_h^{active} \end{pmatrix} \\ &= \begin{pmatrix} -K_a & 0 & -B_a & 0 \\ 0 & -K_h & 0 & -B_h \end{pmatrix} \begin{pmatrix} \theta_a \\ \theta_h \\ \omega_a \\ \omega_h \end{pmatrix} + \begin{pmatrix} -P_a & 0 & -P_h & 0 \\ 0 & -D_a & 0 & -D_h \end{pmatrix} \begin{pmatrix} \theta_{a\Delta} \\ \theta_{h\Delta} \\ \omega_{a\Delta} \\ \omega_{h\Delta} \end{pmatrix} \\ &\equiv \mathbf{T}_p \begin{pmatrix} \theta_a \\ \theta_h \\ \omega_a \\ \omega_h \end{pmatrix} + \mathbf{T}_a \begin{pmatrix} \theta_{a\Delta} \\ \theta_{h\Delta} \\ \omega_{a\Delta} \\ \omega_{h\Delta} \end{pmatrix}. \end{aligned} \tag{A.5}$$

The lower subscript Δ represents the time delay, e.g., $x_\Delta(t) = x(t - \Delta)$. For each joint, elastic and viscosity coefficients are denoted by K_j and B_j, respectively, for $j = \{a, h\}$. P_j and D_j are the proportional and derivative gains of the active neural feedback controller. K_j and P_j are defined by the total mass m of the double pendulum and the distance between the ankle joint to the center of mass position of the double pendulum under a fully extended hip joint condition, as follows.

$$K_j = k_j mgh, \tag{A.6}$$

$$P_j = p_j mgh, \tag{A.7}$$

$$m = m_L + m_{HAT}, \tag{A.8}$$

$$h = \frac{h_L m_L + (l_L + h_{HAT}) m_{HAT}}{m}, \tag{A.9}$$

where k_j and p_j are non-dimensional parameters, K_j and P_j.

Appendix B: Posture estimation of the upper body and lower limbs

In this study, lengths and postures of the upper body and lower extremities were estimated from the markers attached to the characteristic points on a subject's body using the following method.

According to Winter et al. 1998, we considered the human body as a twelve-link system (Pelvis, Trunk 1, Trunk 2, Trunk 3, Trunk 4, Head, L/R-Thigh, L/R-LowerLeg, L/R-Foot), and estimated the center of mass (CoM) position $\mathbf{p}_i[n]$ for each of i = {pelvis, trunk-1, trunk-2, trunk-3, trunk-4, head, l-thigh, r-thigh, l-lower-leg, r-lower-leg, l-foot, r-foot}, with n being the discrete times from the spatiotemporal information of the markers. It should be noted that, in this study, the upper extremities were included in the trunk, and their spatial configuration was not considered. Marker information used for calculating the CoM position of each body segment is shown in Table 3.

$$\mathbf{p}_{\text{pelvis}}[n] = \frac{\mathbf{p}_{\text{L-ASIS}}[n] + \mathbf{p}_{\text{R-SIS}}[n]}{2}, \tag{B.1}$$

$$\mathbf{p}_{\text{trunk-1}}[n] = \frac{\mathbf{p}_{\text{L-IliacCrest}}[n] + \mathbf{p}_{\text{R-IliacCrest}}[n] + \mathbf{p}_{\text{L-ASIS}}[n] + \mathbf{p}_{\text{R-ASIS}}[n]}{4}, \tag{B.2}$$

$$\mathbf{p}_{\text{trunk-2}}[n] = \frac{\mathbf{p}_{\text{L-LowerRib}}[n] + \mathbf{p}_{\text{R-LowerRib}}[n] + \mathbf{p}_{\text{L-IliacCrest}}[n] + \mathbf{p}_{\text{R-IliacCrest}}[n]}{4}, \tag{B.3}$$

$$\mathbf{p}_{\text{trunk-3}}[n] = \frac{2\mathbf{p}_{\text{Xiphoid}}[n] + \mathbf{p}_{\text{L-LowerRib}}[n] + \mathbf{p}_{\text{R-LowerRib}}[n]}{4}, \tag{B.4}$$

$$\mathbf{p}_{\text{trunk-4}}[n] = \frac{\mathbf{p}_{\text{L-Shoulder}}[n] + \mathbf{p}_{\text{R-Shoulder}}[n] + \mathbf{p}_{\text{Xiphoid}}[n]}{3}, \tag{B.5}$$

$$\mathbf{p}_{\text{head}}[n] = \frac{\mathbf{p}_{\text{L-Ear}}[n] + \mathbf{p}_{\text{R-Ear}}[n]}{2}, \tag{B.6}$$

$$\mathbf{p}_{\text{l/r-thigh}}[n] = 0.567 * \mathbf{p}_{\text{l/r-Hip}}[n] + 0.433 * \mathbf{p}_{\text{l/r-Knee}}[n], \tag{B.7}$$

$$\mathbf{p}_{\text{l/r-lower-leg}}[n] = 0.394 * \mathbf{p}_{\text{l/r-Knee}}[n] + 0.606 * \mathbf{p}_{\text{l/r-Ankle}}[n]. \tag{B.8}$$

Time variations of the ankle and hip joint angles during quiet stance for the double inverted pendulum model were obtained by the following four steps: 1) estimation of lengths of the upper body and lower extremities, 2) posture estimation of the lower extremities, 3) estimation of hip joint position under the assumption that the human body is composed of double rigid links, 4) posture estimation of the upper body.

Estimation of lengths of the upper body and lower extremities.

The position of the ankle joint ($\mathbf{p}_{\text{ankle}}[n]$) was estimated as the midpoint of two markers attached to the left and right external malleolus (l/r-Ankle). The position of the hip joint ($\mathbf{p}_{\text{hip}}[n]$) was estimated as the midpoint of two markers attached to the left and right great trochanters (l/r-Hip). The length of the lower extremities (l_{L}) was calculated by performing a time average of the distance between the ankle and hip joints.

$$l_{\text{L}} = \frac{1}{N}\sum_{n=1}^{N} \left|\mathbf{p}_{\text{hip}}[n] - \mathbf{p}_{\text{ankle}}[n]\right|. \tag{B.9}$$

Here, N represents the total number of data. The length of the upper body (l_{HAT}) was calculated by subtracting the length of the lower extremities from the height of the subject.

Posture estimation of the lower extremities

The CoM position of the lower leg ($\mathbf{p}_{\text{lower-leg}}[n]$) was estimated as the midpoint of the CoMs of the left and right lower legs ($\mathbf{p}_{\text{l-lower-leg}}[n]$, $\mathbf{p}_{\text{r-lower-leg}}[n]$). The CoM position of the thigh ($\mathbf{p}_{\text{thigh}}[n]$) was estimated as the midpoint of the CoMs of the left and right thighs ($\mathbf{p}_{\text{l-thigh}}[n]$, $\mathbf{p}_{\text{r-thigh}}[n]$). The CoM position of the lower extremity unit ($\mathbf{p}_{\text{L}}[n]$) was calculated as the internal dividing point between the CoM position of the lower leg and that of the thigh.

$$\mathbf{p}_{\text{L}}[n] = \frac{m_{\text{lower-leg}}\mathbf{p}_{\text{lower-leg}}[n] + m_{\text{thigh}}\mathbf{p}_{\text{thigh}}[n]}{m_{\text{lower-leg}} + m_{\text{thigh}}}. \tag{B.10}$$

where, $m_{\text{lower-leg}}$ and m_{thigh} represent the masses of the lower legs and thighs, respectively (See Table B.6 for details). We defined the inclination angle of the line connecting the ankle joint and the CoM of the lower extremities from the vertical line as the posture of the lower extremity unit ($\varphi_{\text{L}}[n]$).

$$\varphi_{\text{L}}[n] = \frac{\pi}{2} - \tan^{-1}\left(\frac{y_{\text{L}}[n] - y_{\text{ankle}}[n]}{x_{\text{L}}[n] - x_{\text{ankle}}[n]}\right). \tag{B.11}$$

Estimation of hip joint position with the double inverted pendulum model

Time variations of the hip joint position ($\tilde{\mathbf{p}}_{\text{hip}}[n] = (\tilde{x}_{\text{hip}}[n], \tilde{y}_{\text{hip}}[n])$) were estimated using the length and posture of the lower extremities as follows.

$$\tilde{\mathbf{p}}_{\text{hip}}[n] = \begin{pmatrix}\tilde{x}_{\text{hip}}[n]\\ \tilde{y}_{\text{hip}}[n]\end{pmatrix} = \begin{pmatrix}l_{\text{L}}\sin\varphi_{\text{L}}[n]\\ l_{\text{L}}\cos\varphi_{\text{L}}[n]\end{pmatrix}. \tag{B.12}$$

Posture estimation of the upper body

The CoM position of the upper body was estimated as the median point between the CoM positions of the head ($\mathbf{p}_{\text{head}}[n]$), trunk ($\mathbf{p}_{\text{trunk-1}}[n]$, $\mathbf{p}_{\text{trunk-2}}[n]$, $\mathbf{p}_{\text{trunk-3}}[n]$, $\mathbf{p}_{\text{trunk-4}}[n]$), and pelvis ($\mathbf{p}_{\text{pelvis}}[n]$).

$$\mathbf{p}_{\text{HAT}}[n] = \frac{\begin{array}{c}m_{\text{head}}\mathbf{p}_{\text{head}}[n] + m_{\text{trunk-1}}\mathbf{p}_{\text{trunk-1}}[n] + m_{\text{trunk-2}}\mathbf{p}_{\text{trunk-2}}[n]\\ +m_{\text{trunk-3}}\mathbf{p}_{\text{trunk-3}}[n] + m_{\text{trunk-4}}\mathbf{p}_{\text{trunk-4}}[n] + m_{\text{pelvis}}\mathbf{p}_{\text{pelvis}}[n]\end{array}}{m_{\text{head}} + m_{\text{trunk-1}} + m_{\text{trunk-2}} + m_{\text{trunk-3}} + m_{\text{trunk-4}} + m_{\text{pelvis}}}, \tag{B.13}$$

where m_{head}, $m_{\text{trunk-1}}$, $m_{\text{trunk-2}}$, $m_{\text{trunk-3}}$, $m_{\text{trunk-4}}$, and m_{pelvis} represent the masses of the head, trunk, and pelvis, respectively (See Table B.6 for details). We defined the inclination angle of the line connecting the hip joint and the CoM of the upper body from the vertical line as the posture of the upper body ($\varphi_{\text{HAT}}[n]$).

$$\varphi_{\text{HAT}}[n] = \frac{\pi}{2} - \tan^{-1}\left(\frac{y_{\text{HAT}}[n] - y_{\text{hip}}[n]}{x_{\text{HAT}}[n] - x_{\text{hip}}[n]}\right). \tag{B.14}$$

Table B.6. Masses of the body segments. $\widetilde{m}$ is the total body mass (Table 1)

Symbol	Description	Value / Unit
m_{pelvis}	Mass of the pelvis	$\widetilde{m} \times 0.142$ kg
$m_{\text{trunk-1}}$	Mass of Trunk-1	$\widetilde{m} \times 0.078$ kg
$m_{\text{trunk-2}}$	Mass of Trunk-2	$\widetilde{m} \times 0.065$ kg
$m_{\text{trunk-3}}$	Mass of Trunk-3	$\widetilde{m} \times 0.078$ kg
$m_{\text{trunk-4}}$	Sum of Trunk-4 and l/r-Arm masses	$\widetilde{m} \times (0.136+(0.028+0.022)) \times 2$ kg
m_{head}	Mass of the head	$\widetilde{m} \times 0.081$ kg
m_{thigh}	Sum of l/r-Thigh masses	$\widetilde{m} \times 0.100 \times 2$ kg
$m_{\text{lower-leg}}$	Sum of l/r-Lower-Leg masses	$\widetilde{m} \times 0.060 \times 2$ kg

Appendix C: Method for projecting the stable and unstable manifolds of the double inverted pendulum model in the θ_a-θ_h and ω_a-ω_h planes

The equilibrium point of the double inverted pendulum model without active feedback control (off-model) is saddle-type and unstable, and there is a one-dimensional unstable manifold and one-dimensional stable manifold (both corresponding to the in-phase mode), and a two-dimensional stable manifold corresponding to the anti-phase mode in the four-dimensional state space. These manifolds cannot be represented accurately in the θ_a-θ_h and ω_a-ω_h planes. In this study, we visualized these manifolds in the θ_a-θ_h and ω_a-ω_h planes as follows.

Visualization of the one-dimensional manifold

Here, the eigenvector $\mathbf{v}_{1d}$ that spans the one-dimensional manifold is denoted as follows.

$$\mathbf{v}_{1\mathrm{d}} = \begin{pmatrix} \theta_\mathrm{a}^{1\mathrm{d}} \\ \theta_\mathrm{h}^{1\mathrm{d}} \\ \omega_\mathrm{a}^{1\mathrm{d}} \\ \omega_\mathrm{h}^{1\mathrm{d}} \end{pmatrix}. \quad \text{(C.1)}$$

For an arbitrary state point on the one-dimensional manifold, the ratio between the ankle joint angle θ_a and the hip joint angle θ_h always satisfies $\theta_a{:}\theta_h = \theta_a^{1d}{:}\theta_h^{1d}$, regardless of the values of the ankle joint angular velocity ω_a and the hip joint angular velocity ω_h. In the same way, for an arbitrary state point on the one-dimensional manifold, the ratio between the ankle joint angular velocity ω_a and the hip joint angular velocity ω_h always satisfies $\omega_a{:}\omega_h = \omega_a^{1d}{:}\omega_h^{1d}$, regardless of the values of the ankle joint angle θ_a and the hip joint angle θ_h. Therefore, the one-dimensional manifold as the straight line both on the θ_a-θ_h plane and the ω_a-ω_h plane can be visualized using the following equations.

$$\theta_\mathrm{h} = \frac{\theta_\mathrm{h}^{1\mathrm{d}}}{\theta_\mathrm{a}^{1\mathrm{d}}}\theta_\mathrm{a}, \quad \text{(C.2)}$$

$$\omega_\mathrm{h} = \frac{\omega_\mathrm{h}^{1\mathrm{d}}}{\omega_\mathrm{a}^{1\mathrm{d}}}\omega_\mathrm{a}. \quad \text{(C.3)}$$

Visualization of the two-dimensional manifold

Here, the eigenvectors $\mathbf{v}_{2d,a}$ and $\mathbf{v}_{2d,b}$ that span the two-dimensional manifold are denoted as follows.

$$\mathbf{v}_{2\mathrm{d,a}} = \begin{pmatrix} \theta_\mathrm{a}^{2\mathrm{d,a}} \\ \theta_\mathrm{h}^{2\mathrm{d,a}} \\ \omega_\mathrm{a}^{2\mathrm{d,a}} \\ \omega_\mathrm{h}^{2\mathrm{d,a}} \end{pmatrix}, \mathbf{v}_{2\mathrm{d,b}} = \begin{pmatrix} \theta_\mathrm{a}^{2\mathrm{d,b}} \\ \theta_\mathrm{h}^{2\mathrm{d,b}} \\ \omega_\mathrm{a}^{2\mathrm{d,b}} \\ \omega_\mathrm{h}^{2\mathrm{d,b}} \end{pmatrix}. \quad \text{(C.4)}$$

Using real value parameters α and β, the two-dimensional manifold can be described as follows.

$$\begin{pmatrix} \theta_{\mathrm{a}} \\ \theta_{\mathrm{h}} \\ \omega_{\mathrm{a}} \\ \omega_{\mathrm{h}} \end{pmatrix} = \alpha \mathbf{v}_{2\mathrm{d,a}} + \beta \mathbf{v}_{2\mathrm{d,b}} \tag{C.5}$$

For a set of values of (ω_a, ω_h), α and β, which satisfy the equalities for the third and fourth elements in Eq. (C.5), are specified and then corresponding θ_a and θ_h values are determined according to the equalities for the first and second elements in Eq. (C.5) as follows.

$$\begin{pmatrix} \alpha \theta_{\mathrm{a}}^{2\mathrm{d,a}} + \beta \theta_{\mathrm{a}}^{2\mathrm{d,b}} \\ \alpha \theta_{\mathrm{h}}^{2\mathrm{d,a}} + \beta \theta_{\mathrm{h}}^{2\mathrm{d,b}} \end{pmatrix} \tag{C.6}$$

That is, the coordinate point (Eq. (C.6)) on the θ_a-θ_h plane corresponds to one point on the two-dimensional manifold with the set of values of (ω_a, ω_h) (See Fig. 3). In this paper, each ω_a and ω_h is set to either −0.03 (rad/sec) or 0.03 (rad/sec), i.e., the four vertices of the square on the ω_a-ω_h plane ((ω_a, ω_h) = (−0.03, −0.03), (−0.03, 0.03), (0.03, −0.03), (0.03, 0.03)), and plotted on the θ_a-θ_h plane according to Eq. (C.6) as the four points on the two-dimensional stable manifold. The four vertices of the square on the ω_a-ω_h plane are mapped to the four vertices of the parallelogram on the θ_a-θ_h plane by Eq. (C.6), and the inside of the parallelogram in the θ_a-θ_h plane represents the two-dimensional manifold with ω_a and ω_h values from −0.03 to 0.03.

In the same manner, for a set of values of (θ_a, θ_h), α and β, which satisfy the equalities for the first and second elements in Eq. (C.5), are specified and then corresponding ω_a and ω_h values are determined according to the equalities for the third and fourth elements in Eq. (C.5) as follows.

$$\begin{pmatrix} \alpha \omega_{\mathrm{a}}^{2\mathrm{d,a}} + \beta \omega_{\mathrm{a}}^{2\mathrm{d,b}} \\ \alpha \omega_{\mathrm{h}}^{2\mathrm{d,a}} + \beta \omega_{\mathrm{h}}^{2\mathrm{d,b}} \end{pmatrix} \tag{C.7}$$

In this paper, each θ_a and θ_h is set to either −0.02 (rad) or 0.02 (rad), i.e., the four vertices of the square on the θ_a-θ_h plane ((θ_a, θ_h) = (−0.02, −0.02), (−0.02, 0.02), (0.02, −0.02), (0.02, 0.02)), and plotted on the ω_a-ω_h plane according to Eq. (C.7) as the four points on the two-dimensional stable manifold. The four vertices of the square on the θ_a-θ_h plane are mapped to the four vertices of parallelogram on the ω_a-ω_h plane by Eq. (C.7), and the inside of the parallelogram on the ω_a-ω_h plane corresponds to the two-dimensional manifold with the θ_a and θ_h values from −0.02 to 0.02.

Appendix D: Analysis of the double inverted pendulum with continuous active feedback control

In this study, we analyzed the double inverted pendulum model with active feedback control (on-model) using the same method used in the previous study (Suzuki et al. 2012). The on-model is described by the delay differential equation, which includes time delay due to signal processing (Eq. (8)). Here, we consider a solution of Eq. (8) described as follows.

$$\begin{pmatrix} \theta_{\mathrm{a}}(t) \\ \theta_{\mathrm{h}}(t) \\ \omega_{\mathrm{a}}(t) \\ \omega_{\mathrm{h}}(t) \end{pmatrix} = e^{\lambda t} \begin{pmatrix} \theta_{\mathrm{a}}([-\Delta, 0]) \\ \theta_{\mathrm{h}}([-\Delta, 0]) \\ \omega_{\mathrm{a}}([-\Delta, 0]) \\ \omega_{\mathrm{h}}([-\Delta, 0]) \end{pmatrix}. \tag{D.1}$$

Then, Eq. (8) is transformed as

$$\lambda e^{\lambda t} \begin{pmatrix} \theta_{\mathrm{a}}([-\Delta, 0]) \\ \theta_{\mathrm{h}}([-\Delta, 0]) \\ \omega_{\mathrm{a}}([-\Delta, 0]) \\ \omega_{\mathrm{h}}([-\Delta, 0]) \end{pmatrix} = \left(e^{\lambda t}\mathbf{A} + e^{\lambda(t-\Delta)}\mathbf{A}_{\mathrm{a}}\right) \begin{pmatrix} \theta_{\mathrm{a}}([-\Delta, 0]) \\ \theta_{\mathrm{h}}([-\Delta, 0]) \\ \omega_{\mathrm{a}}([-\Delta, 0]) \\ \omega_{\mathrm{h}}([-\Delta, 0]) \end{pmatrix}. \tag{D.2}$$

Except for the trivial zero solution, λ must satisfy the following transcendental equation for Eq. (D.2) to have a solution.

$$\left|\lambda \mathbf{I} - \mathbf{A} - e^{-\lambda\Delta}\mathbf{A}_{\mathrm{a}}\right| = 0. \tag{D.3}$$

In this study, we calculated λ satisfying Eq. (D.3) using the Newton method. Although Eq. (D.3) has an infinite number of roots, we selected and used four dominant solutions, which have the largest values in their real parts, for analysis. If all real parts of λ are negative, then the equilibrium of the on-model is determined as stable. Alternatively, if λ has a positive real part, then the equilibrium is determined as unstable. For each λ, the dynamic mode is determined by using the corresponding eigenvector. We selected λ corresponding to the anti-phase mode, and calculated the eigenfrequency of the anti-phase mode from the imaginary part of λ.
